# Is adaptation involved in bilingual language production? A fresh look at the assumptions motivating potential bilingual-monolingual differences in adaptive control

**DOI:** 10.3758/s13423-024-02503-6

**Published:** 2024-05-07

**Authors:** Giacomo Spinelli, Simone Sulpizio

**Affiliations:** Department of Psychology, University of Milano-Bicocca, Piazza dell’Ateneo Nuovo 1, 20126 Milan, Italy

**Keywords:** Adaptive control, Bilingualism, Bilingual advantage, Cognitive control

## Abstract

One of the hottest debates in psychology—whether bilingual-monolingual differences exist in cognitive control—is at a stalemate. Here we propose that the stalemate could be broken by shifting the research focus from *whether* those differences emerge to *why* they should. We offer an example of this approach by testing the assumption of current theories of language-control associations that adaptive control is involved in bilingualism, specifically language production. Unbalanced Italian-English bilinguals living in the Milan area completed a Stroop task in their L1 and a picture-naming task in their L2. Both tasks involved a manipulation of the proportion of the type of stimuli that are assumed to require control, i.e., incongruent stimuli in the Stroop task (e.g., the word RED written in blue) and pictures with noncognate names in the picture-naming task (e.g., the picture of a horse, whose Italian name, “cavallo,” has a very different pronunciation). Both confirmatory and exploratory analyses showed a clear dissociation between the two tasks, with the Stroop task producing an interactive pattern indicative of adaptive-control involvement and the picture-naming task failing to produce a similar one. These results suggest that adaptive control may not be involved in bilingual language production and, therefore, may not produce bilingual-monolingual differences in cognitive control. It is hoped that this research will inspire a change in the study of language-control associations, pushing future research efforts towards grounding the assumptions for those associations in empirical evidence.

**Supplementary Information:**

The online version contains supplementary material available at 10.3758/s13423-024-02503-6.

## Introduction

Every so often the bilingual-advantage saga—the debate over whether regular experience managing two languages confers bilinguals an advantage in cognitive control relative to monolinguals—features a promising new episode. The audience gasp: Will the advantage convince skeptics *this time*? But in an all-too-predictable pattern, a new challenge arises, and the solution will have to wait for another episode (Bialystok, 2017; Paap, 2022; see also Antoniou, 2019).

Here we propose that part of the reason for the stalemate affecting current theories of language-control associations (e.g., Bialystok, 2017; Bialystok & Craik, 2022; Green & Abutalebi, 2013) is that while much of the debate has focused on the *existence* of bilingual-monolingual differences—the theories’ predictions—little attention has been paid to the *motivation* for potential differences—the theories’ assumptions (but see Blanco-Elorrieta & Caramazza, 2021). However, prioritization of the latter would be necessary: If there was little or no motivation for there being bilingual-monolingual differences in a certain control ability, there would be no good reason to even attempt to find them. If, in contrast, the motivation was solid, such attempts would have a much higher chance of success, and failures would gain informative value (Oberauer & Lewandowsky, 2019).

Based on these considerations, rather than conducting the umpteenth contrast between monolinguals and bilinguals, we focused on the latter to examine a core assumption of current theories of language-control associations: the idea that experience managing two languages involves adaptive (or attentional) control (e.g., Bialystok, 2017; Green & Abutalebi, 2013). Adaptive control refers to the ability to adjust processing selectivity in line with the current goal and context (Braem et al., 2019) and is typically studied in conflict tasks such as the Stroop task (1935), where conflict from an irrelevant but easily processed distractor in incongruent stimuli (e.g., the word RED written in blue) elicits slower and less accurate responses than the absence of such conflict in congruent stimuli (e.g., RED in red). Demonstrating adaptive control is the fact that the magnitude of this congruency effect is modulated, for example, by the congruency status of the previous trial (Gratton et al., 1992) or the proportion of congruent/incongruent trials in the experimental list (Logan & Zbrodoff, 1979).

**Fig. 1:**
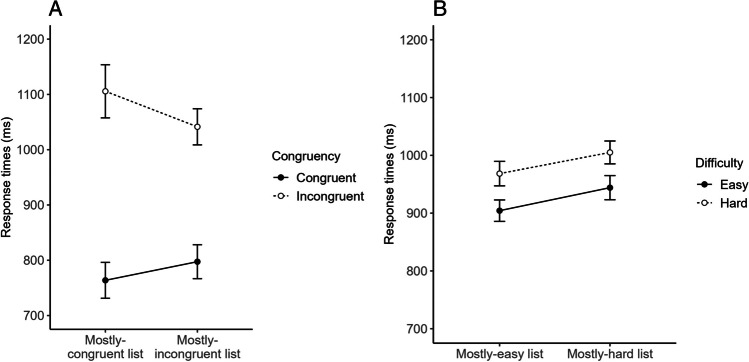
Examples of interactive and additive patterns in conflict (**A**) and non-conflict (**B**) tasks based on Spinelli et al.’s (2019) Experiments 1B and 2, respectively

The Proportion-Congruent (PC) manipulation, in particular, produces an interactive pattern (Fig. 1A) involving a larger congruency effect in a list in which the stimuli are mostly congruent than in a list in which the stimuli are mostly incongruent. This PC effect is typically interpreted as reflecting better ability to proactively prepare for conflict in the mostly-incongruent than the mostly-congruent list, in which, instead, conflict would be handled reactively (i.e., when it occurs) and less efficiently (Braver, 2012). That the PC effect is due to conflict and not to a mere difficulty difference between congruent and incongruent stimuli is demonstrated by the fact that parallel experiments manipulating the proportion of easy and hard stimuli (e.g., high-resolution and low-resolution pictures) tend to produce an *additive* pattern (Fig. 1B). This pattern involves a general slow-down but little or no difficulty effect reduction in a mostly-hard list relative to a mostly-easy list (Spinelli et al., 2019). While this slow-down may reflect a later criterion for response emission in contexts in which hard stimuli prevail (Lupker et al., 1997), it most certainly does not reflect adaptive control because it involves no processing selectivity adjustment.

The assumption that adaptive control is involved in bilingualism implies that there are situations in managing two languages in which performance resembles the interactive pattern but not the additive pattern. Here, we tested that idea by attempting to create such a situation in a linguistic task inspired by the conflict-task literature. In conflict tasks, as noted, conflict from a distractor is assumed to produce the congruency effect and trigger adaptive control. In managing two languages, a similar conflict is assumed to occur when producing the name of a concept in a language—the target language—while ignoring the corresponding name in the other language—the non-target language, which, albeit irrelevant, will be active at the same time (e.g., Green, 1998). This conflict would be especially relevant when the non-target language is the bilingual’s dominant language (i.e., their L1, with the target language being their L2; e.g., Hermans et al., 1998) and the two translation equivalents have very different pronunciations (i.e., they are noncognates; e.g., an Italian-English bilingual saying “horse” while ignoring “cavallo,” its Italian equivalent). On the other hand, when the translation equivalents have similar pronunciations (i.e., they are cognates, e.g., “elephant” and “elefante”), conflict is presumably reduced, as evidenced by the fact that pictures with cognate names are named faster than pictures with noncognate names (Costa et al., 2000; for evidence from other tasks, see Santesteban & Schwieter, 2020).

Although originally named the cognate “facilitation” effect, this effect must include both facilitation and interference unless it is assumed that there is no competition between target and non-target languages (Costa et al., 2000). Indeed, such a competition is a basic premise of models that assume cognitive-control involvement in bilingualism (e.g., Green, 1998; Green & Abutalebi, 2013). The cognate effect thus resembles the congruency effect in conflict tasks, which also involves facilitation and interference (MacLeod, 1991). Further, this effect appears to reflect a structural component of bilingualism (i.e., the constant need to deal with irrelevant information from the non-target language), unlike other effects presumed to involve bilingualism-specific control which have turned out to be somewhat epiphenomenal (e.g., language-switching effects; see Blanco-Elorrieta & Pylkkänen, 2018).

**Fig. 2:**
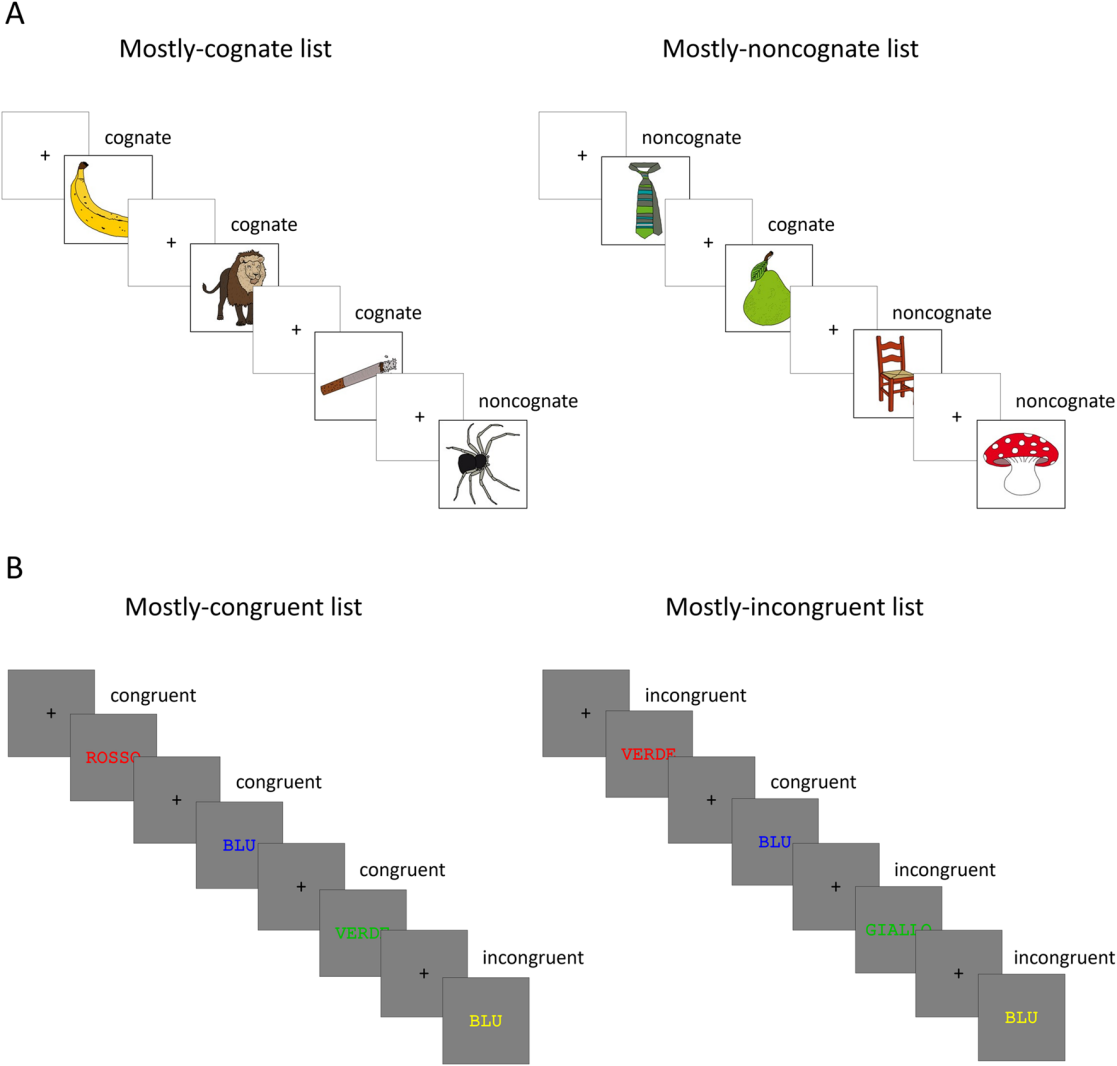
Representation of the composition of the lists used in the L2 picture-naming task (**A**) and the L1 Stroop task (**B**). In the particular counterbalancing represented for the L1 Stroop task (**B**), the colors red and green (and the corresponding Italian names, “rosso” and “verde”) form the inducer subset and are always congruent in the mostly-congruent list and always incongruent in the mostly-incongruent list. The colors blue and yellow (and the corresponding Italian names, “blu” and “giallo”) form the diagnostic subset and are congruent and incongruent in equal proportions in both lists. See the online version of this article for colors

Based on these considerations, we elected the cognate effect in L2 picture naming as a bilingual analog of the congruency effect in conflict tasks and manipulated cognate/noncognate proportion as in conflict tasks, i.e., we created a list in which the pictures were mostly cognate and another in which the pictures were mostly noncognate. Doing so allowed us to contrast two hypotheses: (1) the (alternative) hypothesis that, similar to conflict tasks, adaptive control would be regulated proactively in the mostly-noncognate list to reduce non-target-language conflict and reactively in the mostly-cognate list to deal with that conflict only when it occurs, resulting in a PC-like effect (Fig. 1A); (2) the (null) hypothesis that, similar to non-conflict tasks, a later response criterion would be set in the mostly-noncognate than the mostly-cognate list but no adaptive control would be involved, resulting in an additive pattern (Fig. 1B). The first hypothesis would support a core assumption of theories of language-control associations, i.e., that adaptive control is involved in bilingualism, whereas the second hypothesis would challenge it.

As a manipulation check, a Stroop task in participants’ L1 was also included with a PC manipulation parallel to that used in the L2 picture-naming task. The expectation was for a PC effect to emerge with the critical stimuli (see below), suggesting that our participants were able to engage adaptive control, as participants in this type of experiments typically are (Spinelli & Lupker, 2023).

## Method

### Participants

The sample size needed for a power of .80 to obtain the key interaction effect between cognate status and list type was calculated with G*Power 3.1 (Faul et al., 2009) using the smallest of the PC effects controlled for non-conflict processes reported by Spinelli and Lupker (2023) for a series of color-word Stroop experiments, ${\eta }_{p}^{2}$ = .276. Although the minimum sample size suggested by the analysis was 24, we aimed to reach a sample size comparable to that used in Spinelli and Lupker’s (2023) experiments, i.e., 48 participants. Participants were recruited by advertising the study in social media groups associated with, and classes offered at, the University of Milano-Bicocca, through the university’s participant pool, and from the experimenters’ social circles (see recruitment tools in the Online Supplementary Materials (OSM)). Participants received course credits for their participation. To participate, volunteers were required to consider Italian to be their native (or one of their native) language(s), to have normal or corrected-to-normal vision and hearing, to be between 18 and 45 years old, and to pass an English pre-screening test (see below). 129 participants completed the pre-screening test. Of these, 71 passed it, 60 came to the lab to complete the study, and 48 remained after exclusions (see below). Of the final sample, 36 identified themselves as female, 11 as male, and one as non-binary, with 22.81 years of age on average (*SD* = 3.53, range = 18–36); 40 reported knowing a third language besides Italian and English and 20 a fourth language, although their proficiency, immersion, and dominance in those languages (as calculated using the Language History Questionnaire (LHQ3); Li et al., 2019) was lower than those reported for Italian or English on average; all were born in Italy except two who came to live in Italy during childhood; and all resided in Italy except one who resided in Switzerland. We report additional information on Italian (L1) and English (L2), the two languages involved in the study, in Table 1.

**Table 1:** Characteristics of Italian (L1) and English (L2) for our participants

	Italian (L1)			English (L2)		
Characteristic	Mean	SD	Range	Mean	SD	Range
Proficiency	.95	.07	.79–1	.79	.09	.57–1
Immersion	.90	.05	.63–.96	.72	.06	.56–.86
Dominance	.61	.05	.53–.73	.44	.07	.32–.63
Pre-screening score				20.85	1.79	18–25
Lexical fluency				62.96	7.72	48–81

*Note.* Proficiency, immersion, and dominance are aggregated scores ranging from 0 to 1 calculated using the formulas in the LHQ3 (explained in the Online Supplementary Materials along with the corrections we applied; note that because of those corrections, dominance for the final sample could not be calculated for Italian in six cases and for English in one case). The pre-screening score is the sum of correct responses to the 25 questions included in Cambridge’s online test for adult learners of English, and lexical fluency is the number of correct L1-to-L2 translations provided for 90 words (see *Materials and procedure*)

### Materials and procedure

#### Pre-screening session

Participants were pre-screened using Cambridge’s online test for adult learners of English (https://www.cambridgeenglish.org/test-your-english/general-english/). The test provides an English proficiency estimate within the Common European Framework of Reference for Languages (CEFRL). To pass the test, participants were required to perform at an estimated B2 CEFRL level (see OSM for further details). To participate in the pre-screening test and the subsequent lab session, participants expressed their informed consent. The study was approved by the university’s Ethics Board (protocol RM-2021-445).

#### Lab session

Participants who passed the pre-screening test were invited to participate in the lab session, which comprised a language background questionnaire, an L2 picture-naming task, an L1 Stroop task, and an L1-to-L2 translation task, in this order. All instructions were given in Italian. The whole session took about 2.5 h to complete.

##### Language background questionnaire

To assess participants’ language background, we used the Language History Questionnaire 3.0 (LHQ3; Li et al., 2019), a validated tool to measure, by self-report, several aspects of the bilingual experience such as Age of Acquisition (AoA), proficiency, and patterns of language use. The English version of the LHQ3 was translated into Italian and re-created using the Jotform (https://www.jotform.com/) survey services.

##### L2 picture-naming task

Of the colored drawings in the MultiPic dataset (Duñabeitia et al., 2018), 96 with cognate and 96 with noncognate English and Italian names were selected based on the results of a pilot study (described in full, along with the selection process, in the OSM; for the most relevant characteristics, see Table 2). Each set was split into four subsets of 24 stimuli, roughly matched on the most relevant characteristics reported in Table 2, which were used to create the mostly-cognate and the mostly-noncognate lists. Namely, the mostly-cognate list included three subsets of the cognate set and one subset of the noncognate set (i.e., there were 72 (75%) cognate pictures and 24 (25%) noncognate pictures); the mostly-noncognate list included the fourth subset of the cognate set and the other three subsets of the noncognate set (i.e., there were 24 (25%) cognate pictures and 72 (75%) noncognate pictures; note that these percentages are typical of those used in proportion-congruent manipulations in conflict tasks, see, e.g., Braem et al., 2019). The assignment of subsets to list types was counterbalanced across participants, as was the order of presentation of the list types. The order of presentation of the stimuli within each block was randomized. For a representation of the composition of the two lists, see Fig. 2A.

**Table 2:** Characteristics of the cognate and noncognate stimuli used in the L2 picture-naming task

	Cognate			Noncognate		
Characteristic	Mean	SD	Range	Mean	SD	Range
Visual complexity (picture)	2.46	.46	1.19–3.45	2.36	.32	1.42–3.39
Number of syllables (word)	2.18	.79	1–3	1.50	.66	1–2
Zipf frequency (word)	4.08	.42	3.12–5.02	4.13	.55	.70–5.09
Phonological similarity with Italian (word)	78.41	9.86	60.48–99.64	3.84	3.11	.88–19.72

*Note.* Visual complexity was extracted from the MultiPic norms (Duñabeitia et al., 2018) and is expressed on a 1–5 scale. Number of syllables was extracted from N-Watch (Davis, 2005). Zipf frequency was extracted from Subtlex-UK (van Heuven et al., 2014). Phonological similarity with Italian was extracted from our pilot study and is expressed on a 0–100 scale (see Online Supplementary Materials, specifically, the subsection *Online session* of the section *Materials and procedure* of the pilot study)

Each trial began with a fixation symbol (+) presented for 250 ms followed by the picture presented for 3,000 ms or until response. All pictures were 300-pixel wide and 300-pixel high. Participants were instructed to name the picture in English, their L2, as quickly as possible with the name that they thought was the most appropriate. They were told to speak clearly, without hesitations, and not to worry excessively about their Italian accent. There was a self-paced pause between the two lists. Prior to the experiment, participants completed a practice session with six “neutral” pictures not clearly classifiable as either cognate or noncognate (see OSM). DMDX (Forster & Forster, 2003) was used to program the task.

##### **L1 Stroop task** 

Two subsets of colors (red and green vs. blue and yellow) and the corresponding Italian names (ROSSO and VERDE vs. BLU and GIALLO) were used to create the mostly-congruent and mostly-incongruent lists. One subset was used as the “inducer” subset and included either congruent stimuli only (in the mostly-congruent list) or incongruent stimuli only (in the mostly-incongruent list), whereas the other subset was used as the “diagnostic” subset and included congruent and incongruent stimuli in equal proportions (in both lists). Specifically, the mostly-congruent list included 96 congruent stimuli from one subset, the inducer subset (e.g., the red/green subset), and 48 congruent and 48 incongruent stimuli from the other subset, the diagnostic subset (e.g., the blue/yellow subset; i.e., in total, there were 144 (75%) congruent stimuli and 48 (25%) incongruent stimuli); the mostly-incongruent list included 96 incongruent stimuli from the subset used as the inducer subset in the mostly-congruent list (e.g., the red/green subset) and 48 congruent and 48 incongruent stimuli from the subset used as the diagnostic subset in the mostly-congruent list (e.g., the blue/yellow subset; i.e., in total, there were 48 (25%) congruent stimuli and 144 (75%) incongruent stimuli). The assignment of subsets to the inducer versus diagnostic type of subset was counterbalanced across participants, as was the order of presentation of the list types (but note that this order was always compatible with that used in the L2 picture-naming task, e.g., participants presented with the mostly-cognate list first in that task were always presented with the mostly-congruent list first in this task). This splitting of stimuli into two subsets is the recommended procedure for measuring adaptive control in Stroop-like tasks (Braem et al., 2019). The order of presentation of the stimuli within each block was randomized. For a representation of the composition of the two lists, see Fig. 2B.

Each trial began with a fixation symbol (+) presented for 250 ms followed by the stimulus presented in Courier New pt. 14 font for 2,000 ms or until response. All stimuli appeared against a medium-grey background. Participants were instructed to name the color in Italian, their L1, as quickly and as accurately as possible. There was a self-paced pause between the two lists. Prior to the experiment, participants completed a practice session including eight neutral stimuli (i.e., #####). DMDX (Forster & Forster, 2003) was used to program the task.

##### L2-to-L1 translation task

To assess participants’ L2 lexical fluency, we used an L1-to-L2 translation task comprising 30 high-frequency, 30 medium-frequency, and 30 low-frequency Italian words, all of which had one (in the case of one of the words, two) acceptable English translation(s) according to Word Reference (https://www.wordreference.com/) and none of which had been involved in the previous tasks or were Italian-English cognates (Sulpizio et al., 2019). Participants completed this task with no time limit in an Excel spreadsheet in which the words appeared one above the other in a fixed order (from high to low frequency).

#### Data analysis

Here we report the appropriate confirmatory analyses to test the idea that adaptive control is involved in bilingual language production as it is in conflict tasks. As such, those analyses focus on the group-level results for L2 picture naming and L1 Stroop separately. Exploratory analyses examining individual-level associations between linguistic variables and performance on L2 picture naming or L1 Stroop, and between performance across the two tasks, are reported in the OSM.

For both L2 picture naming and L1 Stroop, the waveforms of responses were manually inspected with CheckVocal (Protopapas, 2007) to determine the accuracy of the response and the correct placement of timing marks. For L2 picture naming, there was some leniency concerning the participant’s pronunciation, but a response was considered correct only if it matched the response that we expected based on the results of our pilot study. (We used a similar criterion when scoring responses to the L1-to-L2 translation task. That is, we were lenient with incorrect spellings (e.g., “rackoon” instead of “raccoon”), but a response was considered correct only if it matched the acceptable response for that word.) Prior to the analyses, invalid trials due to technical failures, responses faster than 300 ms, and null responses (1,209 observations for L2 picture naming and 147 for L1 Stroop) were discarded. Prior to the latency analyses, incorrect responses (1,497 observations for L2 picture naming and 246 for L1 Stroop) were also discarded. Further, in line with current recommendations (Braem et al., 2019), only stimuli from the diagnostic subset were used in the Stroop task analyses. After discarding invalid and incorrect responses, 12 participants contributed fewer than 70% of their original observations in the L2 picture-naming task. Those participants (whose original observations were 2,304 for L2 picture naming and 4,608 for L1 Stroop) were removed from the analyses—a criterion determined a priori in line with previous work (Spinelli et al., 2020; Spinelli & Lupker, 2023)—leaving, as noted, 48 participants in the final sample. Analyses with the full sample, reported in the OSM, produced a similar pattern of results.

All analyses were conducted in R version 4.2.2 (R Core Team, 2022). R-default treatment contrasts were changed to sum-to-zero contrasts (i.e., contr.sum) to help interpret lower-order effects in the presence of higher-order interactions. Separate analyses were conducted for L2 picture naming and L1 Stroop. For both tasks, linear mixed-effects models were used to fit trial-level response times (RTs) and generalized linear mixed-effects models were used to fit trial-level accuracy specifying a binomial distribution with a logit link between fixed effects and the dependent variable. Also, for both tasks, the model included random intercepts for participants and target stimuli. Analyses with the maximal random structure allowed by the data (Bates et al., 2015), reported in the OSM, produced a similar pattern of results. For L2 picture naming, the fixed effects were Cognate Status (cognate vs. noncognate) and List Type (mostly-cognate vs. mostly-noncognate); for L1 Stroop, they were Congruency (congruent vs. incongruent) and List Type (mostly-congruent vs. mostly-incongruent). Analyses with List Type Order (i.e., the order in which the two list types in the two tasks were administered) as an additional fixed effect produced a similar pattern of results, although in the Stroop task, in addition to an overall practice effect, the order in which participants received the lists was found to modulate the PC effect in the RTs (for details, see the OSM). However, both participants who received the mostly-congruent list first and those who received the mostly-incongruent list first showed a PC effect, a testament to the robustness of this effect.

Going back to the present analyses, for RTs, i.e., the most relevant dependent measure for the key patterns emerging in conflict and non-conflict tasks (Spinelli et al., 2019), we also obtained the best-fitting model using backward selection. Further, to quantify the evidence for/against the key interaction between List Type and Cognate Status (for L2 picture naming)/Congruency (for L1 Stroop), we fit two Bayesian models—an RT model with that interaction, interpreted as the alternative hypothesis *H*_*1*_, and an RT model without that interaction, interpreted as the null hypothesis *H*_*0*_. The contrast between the two models yielded *BF*_10_, with values above 1 representing evidence for the presence of the interaction and values below 1 representing evidence for the absence of the interaction (values around 1 would represent no real evidence for either hypothesis). The functions and packages used are reported in the OSM.

## Results

### L2 picture-naming task

**Fig. 3:**
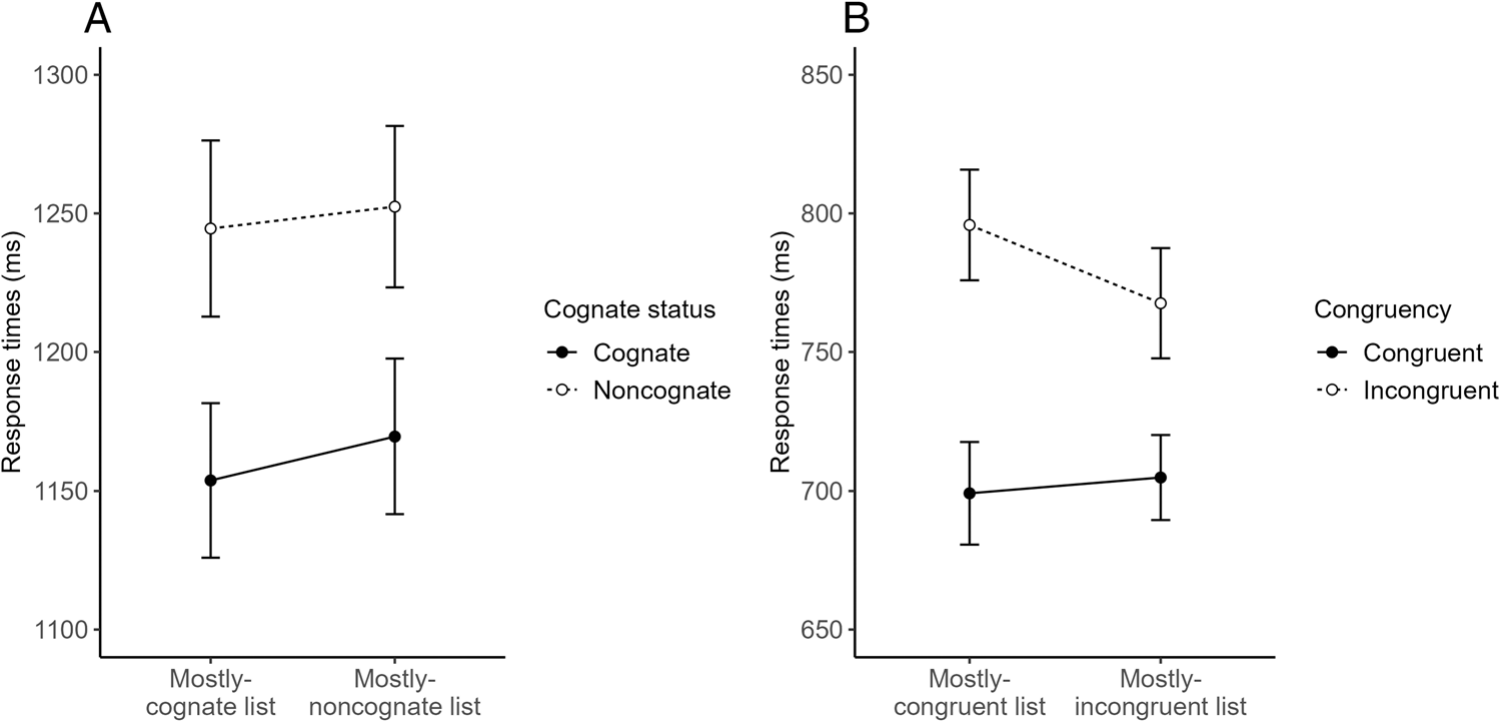
Mean participant-based response times (and corresponding 95% confidence intervals calculated using Cousineau’s (2019) method) in the L2 picture-naming task (**A**) and the L1 Stroop task (**B**)

The mean participant-based RTs are presented in Fig. 3A and in Table 3 along with mean error rates. Full results from the RT and accuracy models are reported in Table 4. As Table 4 shows, whereas no effect reached significance in the accuracy data, in the RTs there was a significant main effect of Cognate Status reflecting, as expected, faster responses to cognate than noncognate pictures, and a marginal (*p* = .050) main effect of List Type reflecting a numerical tendency for faster responses in the mostly-cognate than the mostly-noncognate list. Most importantly, the two effects did not interact: The cognate effects in the mostly-cognate list (91 ms) and the mostly-noncognate list (82 ms) were equivalent. Overall, this type of pattern resembles the additive pattern typical of non-conflict tasks (compare Fig. 3A with Fig. 1B). Indeed, using backward selection, the best-fitting model was the additive one, a model in which the effects of Cognate Status and List Type were both significant (*p* = .006 and *p* = .048, respectively; see the OSM for full results). The Bayes factor, *BF*_10_ = .06 ±6.47%, also favored the additive model over the interactive one.

**Table 3:** Mean participant-based response times and percentage error rates (and corresponding 95% confidence intervals calculated using Cousineau’s (2019) method) in the L2 picture-naming task

	Response times		Error rates	
Cognate status	Mostly-cognate list	Mostly-noncognate list	Mostly-cognate list	Mostly-noncognate list
Cognate	1154 [1126, 1182]	1170 [1142, 1198]	10.77 [9.24, 12.29]	10.99 [8.70, 13.29]
Noncognate	1245 [1213, 1276]	1252 [1223, 1282]	12.03 [8.75, 15.30]	12.38 [10.44, 14.32]
Cognate effect	91	82	1.26	1.39

**Table 4:** Variances and standard deviations for the random effects and coefficients, standard errors, statistics, and probability values for the fixed effects used in the models of response times and accuracy in the L2 picture-naming task

	Response times				Accuracy			
Random effects	*Variance*	*SD*			*Variance*	*SD*		
Participant (intercept)	19765.68﻿	140.59			.266	.516		
Picture (intercept)	33685.08	183.53			1.358	1.165		
Fixed effects	*β*	*SE*	*t*	*p*	*β*	*SE*	*z*	*p*
Intercept	1227.09	24.67	49.74	< .001	2.547	.125	20.33	< .001
Cognate Status	-39.38	14.02	-2.81	.005	.116	.097	1.20	.230
List Type	-8.92	4.56	-1.96	.050	.026	.041	.64	.525
Cognate Status × List Type	-3.23	4.56	-.71	.478	-.003	.041	-.07	.945

The accuracy coefficients are in the logit scale, not in the response scale

### L1 Stroop task

The mean participant-based RTs are presented in Fig. 3B and in Table 5 along with mean error rates. Full results from the RT and accuracy models are reported in Table 6. As Table 6 shows, in the accuracy model, the only significant effect was that of Congruency, reflecting, as expected, more accurate responses to congruent than incongruent stimuli. In the RTs, on the other hand, there was a main effect of Congruency reflecting, as expected, faster responses to congruent than incongruent stimuli overall, a main effect of List Type reflecting slower responses in the mostly-congruent than the mostly-incongruent list overall, and an interaction between the two reflecting, as expected, a larger congruency effect in the mostly-congruent list (97 ms) than in the mostly-incongruent list (63 ms). Note that this pattern was mainly driven by the incongruent stimuli being slower in the mostly-congruent than the mostly-incongruent list, *β* = 28.13, *SE* = 4.28, *z* = 6.57, *p* < .001 (there was no simple main effect of List Type for congruent stimuli, *β* = -5.83, *SE* = 4.23, *z* = -1.38, *p* = .168). Overall, this type of pattern replicates the interactive pattern typical of conflict tasks (compare Fig. 3B with Fig. 1A). The backward selection procedure confirmed that the interactive model was the best-fitting model, save for the elimination of the random effect of target color (note the small amount of variance associated with it in the model reported in Table 6; see the OSM for full results of the best-fitting model). Indeed, the Bayes Factor, *BF*_10_ = 290,657.5 ±10.74%, strongly favored the interactive model over the additive one.

**Table 5:** Mean participant-based response times and percentage error rates (and corresponding 95% confidence intervals calculated using Cousineau’s (2019) method) in the L1 Stroop task

	Response times		Error rates	
Congruency	Mostly-congruent list	Mostly-incongruent list	Mostly-congruent list	Mostly-incongruent list
Congruent	699 [681, 718]	705 [690, 720]	.30 [.01, .60]	.30 [-.03, .64]
Incongruent	796 [776, 816]	768 [748, 787]	3.23 [1.89, 4.57]	2.06 [1.20, 2.92]
Congruency effect	97	63	2.93	1.76

**Table 6:** Variances and standard deviations for the random effects and coefficients, standard errors, statistics, and probability values for the fixed effects used in the models of response times and accuracy in the L1 Stroop task

	Response times				Accuracy			
Random effect	*Variance*	*SD*			*Variance*	*SD*		
Participant (intercept)	11414.40	106.84			.304	.551		
Color (intercept)	6.52	2.55			< .001	< .001		
Fixed effect	*β*	*SE*	*t*	*p*	*β*	*SE*	*z*	*p*
Intercept	741.47	15.55	47.69	< .001	4.861	.170	28.64	< .001
Congruency	-39.51	1.50	-26.27	< .001	1.082	.140	7.74	< .001
List Type	5.58	1.50	3.71	< .001	-.116	.140	-.83	.405
Congruency × List Type	-8.49	1.50	-5.64	< .001	.119	.140	.85	.396

The accuracy coefficients are in the logit scale, not in the response scale

## Discussion

While current theories of language-control associations (e.g., Bialystok, 2017; Green & Abutalebi, 2013) assume that experience managing two languages, particularly in language production, involves adaptive control, that assumption has thus far gone untested. Here we filled this gap by applying a typical manipulation used to demonstrate adaptive control in conflict tasks such as the Stroop task—i.e., the proportion of congruent/incongruent stimuli in a list—to a purely linguistic task involving L2 naming of cognate and noncognate pictures presented with unbalanced proportions in a list. The results of the confirmatory group-level analyses showed a clear dissociation between the two tasks: While the Stroop task produced the interactive pattern typical of conflict tasks, suggesting adaptive-control involvement (i.e., a larger congruency effect in the mostly-congruent than the mostly-incongruent list), its linguistic analog produced the additive pattern typical of non-conflict tasks, suggesting no adaptive-control involvement (i.e., a cognate effect and overall slower responses in the mostly-noncognate list, i.e., the more difficult type of list). These key patterns were robust across several analysis procedures, and exploratory analyses revealed no individual-level predictors modulating them.

There are, of course, a few potential objections to the conclusion that bilingual language production does not involve adaptive control. The first, already addressed in the *Introduction*, concerns whether the paradigm we used can be reasonably presumed to engage such control—wouldn’t, for example, a language-switching paradigm be more appropriate? Although such paradigms indubitably involve some form of control, that control is likely neither language-specific (Festman & Schwieter, 2015) nor ecologically valid (Blanco-Elorrieta & Pylkkänen, 2018). In contrast, the cognate effect in a simple picture-naming task such as ours seems to capture a structural component of bilingualism, i.e., the fact that every time a bilingual speaks, they must select a word from the target language and avoid selecting its translation equivalent in the non-target language.

Second, there could have been differences between the Stroop task and the picture-naming task we used (other than the purely linguistic nature of the latter) explaining the dissociation those tasks produced. One such difference is the stimulus set size, which was much smaller in the Stroop task (four colors) than in the picture-naming task (96 pictures per list). Challenging the idea that stimulus set size might matter, however, is the fact that Stroop tasks with stimulus sets even larger than in the present picture-naming task produced the typical interactive pattern (Spinelli et al., 2019). Another difference is that while in our Stroop task the distractor (i.e., the word) was explicitly presented, in our picture-naming task the distractor (i.e., the translation equivalent) was not. However, adaptive control has been demonstrated with internally represented distractors (Kiyonaga & Egner, 2014) and even without awareness of their presence (Desender et al., 2013). Further, that translation equivalents are activated implicitly is part of the normal bilingual experience. Therefore, if adaptive control is normally involved in bilingualism, it should have emerged in this type of situation.

Third and finally, our results could be restricted to the type of population that we sampled—i.e., bilinguals who, despite being relatively proficient in their L2, are immersed in a predominantly L1 environment (for the importance of bilinguals’ social dynamics, see Titone & Tiv, 2023). While we cannot rule out this possibility, note that this type of bilingual profile is likely the most common one for bilinguals, at least in Europe. Further, although bilingual profiles may vary, adaptive control has been proposed as a unifying framework for understanding those profiles (e.g., Green & Abutalebi, 2013). It would seem to follow that evidence for this form of control could be produced by any type of bilingual.

A relevant question that the idea that adaptive control is not involved in bilingual language production would raise, of course, is what control process would handle the competition between languages that bilinguals would seem to face when speaking? As originally proposed by Costa et al. (2000), a language-specific selection mechanism may, on one hand, prevent that competition from arising; on the other hand, it may allow for phonological overlap among cognate translation equivalents to speed up naming, thus producing the cognate effect. That is, cross-language competition, a control need inherent in bilingual language production, would be handled internally and would involve no regular exercise of domain-general processes such as adaptive control.

The crucial implication is that adaptive control would be used no more often by bilinguals than by monolinguals, and would thus not be the key ingredient producing potential bilingual-monolingual differences in cognitive control as has been assumed thus far.^1^ These conclusions need, of course, corroboration from different paradigms, samples, and research groups. Nor do we intend to use these conclusions to dismiss the whole bilingual-advantage enterprise. Our intent, instead, is to invite a rethinking of theories of language-control associations and a shift in research focus from the *existence* of bilingual-monolingual differences to the *motivation* for those potential differences. Without this shift, borrowing Hartsuiker’s (2015) analogy, bilingual-advantage research would be no different than going on a treasure hunt with an unreliable map. Let’s first make sure we have the right map.

## Supplementary Information

Below is the link to the electronic supplementary material.

Supplementary file1 (DOCX 956 KB)

## Data Availability

The datasets analyzed in the present study are available on the Open Science Framework at https://osf.io/chj58/.

## Footnotes

1 Note that albeit bilingual-monolingual differences in adaptive-control effects *have* been reported, the empirical record is, as usual, mixed (cf., e.g., Grundy et al., 2017, and Paap et al., 2019).

## References

[CR1] Antoniou, M. (2019). The advantages of bilingualism debate. *Annual Review of Linguistics*, *5*, 395–415.

[CR2] Bates, D., Kliegl, R., Vasishth, S., & Baayen, H. (2015). Parsimonious mixed models. *ArXiv*, 1506.04967. 10.48550/arXiv.1506.04967

[CR3] Bialystok, E. (2017). The bilingual adaptation: How minds accommodate experience. *Psychological Bulletin,**143*(3), 233–262. 10.1037/bul0000099 28230411 10.1037/bul0000099 PMC5324728

[CR4] Bialystok, E., & Craik, F. I. M. (2022). How does bilingualism modify cognitive function? Attention to the mechanism. *Psychonomic Bulletin & Review,**29*(4), 1246–1269. 10.3758/s13423-022-02057-5 35091993 10.3758/s13423-022-02057-5

[CR5] Blanco-Elorrieta, E., & Caramazza, A. (2021). On the need for theoretically guided approaches to possible bilingual advantages: an evaluation of the potential loci in the language and executive control systems. *Neurobiology of Language,**2*(4), 452–463. 10.1162/nol_a_00041 37214630 10.1162/nol_a_00041 PMC10158579

[CR6] Blanco-Elorrieta, E., & Pylkkänen, L. (2018). Ecological validity in bilingualism research and the bilingual advantage. *Trends in Cognitive Sciences,**22*(12), 1117–1126. 10.1016/j.tics.2018.10.001 30449317 10.1016/j.tics.2018.10.001

[CR7] Braem, S., Bugg, J. M., Schmidt, J. R., Crump, M. J. C., Weissman, D. H., Notebaert, W., & Egner, T. (2019). Measuring adaptive control in conflict tasks. *Trends in Cognitive Sciences,**23*(9), 769–783. 10.1016/j.tics.2019.07.002 31331794 10.1016/j.tics.2019.07.002 PMC6699878

[CR8] Braver, T. S. (2012). The variable nature of cognitive control: a dual mechanisms framework. *Trends in Cognitive Sciences,**16*(2), 106–113. 10.1016/j.tics.2011.12.010 22245618 10.1016/j.tics.2011.12.010 PMC3289517

[CR9] Costa, A., Caramazza, A., & Sebastian-Galles, N. (2000). The cognate facilitation effect: Implications for models of lexical access. *Journal of Experimental Psychology: Learning, Memory, and Cognition,**26*(5), 1283–1296. 10.1037/0278-7393.26.5.1283 11009258 10.1037//0278-7393.26.5.1283

[CR10] Cousineau, D. (2019). Correlation-adjusted standard errors and confidence intervals for within-subject designs: A simple multiplicative approach. *The Quantitative Methods for Psychology*, *15*(3), 226–241. 10.20982/tqmp.15.3.p226

[CR11] Davis, C. J. (2005). N-Watch: A program for deriving neighborhood size and other psycholinguistic statistics. *Behavior Research Methods,**37*(1), 65–70. 10.3758/bf03206399 16097345 10.3758/bf03206399

[CR12] Desender, K., Van Lierde, E., & Van den Bussche, E. (2013). Comparing conscious and unconscious conflict adaptation. *PLoS ONE,**8*(2), e55976. 10.1371/journal.pone.0055976 23405242 10.1371/journal.pone.0055976 PMC3566123

[CR13] Duñabeitia, J. A., Crepaldi, D., Meyer, A. S., New, B., Pliatsikas, C., Smolka, E., & Brysbaert, M. (2018). MultiPic: A standardized set of 750 drawings with norms for six European languages. *Quarterly Journal of Experimental Psychology,**71*(4), 808–816. 10.1080/17470218.2017.1310261 10.1080/17470218.2017.1310261 28326995

[CR14] Faul, F., Erdfelder, E., Buchner, A., & Lang, A.-G. (2009). Statistical power analyses using G*Power 3.1: Tests for correlation and regression analyses. *Behavior Research Methods*, *41*(4), 1149–1160. 10.3758/brm.41.4.1149 10.3758/BRM.41.4.1149 19897823

[CR15] Festman, J., & Schwieter, J. W. (2015). Behavioral measures of language control. In J. W. Schwieter (Ed.), *The Cambridge handbook of bilingual processing* (pp. 527–547). Cambridge, UK: Cambridge University Press. 10.1017/cbo9781107447257.023

[CR16] Forster, K. I., & Forster, J. C. (2003). DMDX: A Windows display program with millisecond accuracy. *Behavior Research Methods, Instruments, & Computers,**35*(1), 116–124. 10.3758/bf03195503 10.3758/bf03195503 12723786

[CR17] Gratton, G., Coles, M. G. H., & Donchin, E. (1992). Optimizing the use of information: Strategic control of activation of responses. *Journal of Experimental Psychology: General,**121*(4), 480–506. 10.1037/0096-3445.121.4.480 1431740 10.1037//0096-3445.121.4.480

[CR18] Green, D. W. (1998). Mental control of the bilingual lexico-semantic system. *Bilingualism: Language and Cognition*, *1*(2), 67–81. 10.1017/s1366728998000133

[CR19] Green, D. W., & Abutalebi, J. (2013). Language control in bilinguals: The adaptive control hypothesis. *Journal of Cognitive Psychology,**25*(5), 515–530. 10.1080/20445911.2013.796377 25077013 10.1080/20445911.2013.796377 PMC4095950

[CR20] Grundy, J. G., Chung-Fat-Yim, A., Friesen, D. C., Mak, L., & Bialystok, E. (2017). Sequential congruency effects reveal differences in disengagement of attention for monolingual and bilingual young adults. *Cognition,**163*, 42–55. 10.1016/j.cognition.2017.02.010 28273520 10.1016/j.cognition.2017.02.010 PMC5398762

[CR21] Hartsuiker, R. J. (2015). Why it is pointless to ask under which specific circumstances the bilingual advantage occurs. *Cortex,**73*, 336–337. 10.1016/j.cortex.2015.07.018 26303278 10.1016/j.cortex.2015.07.018

[CR22] Hermans, D., Bongaerts, T., De Bot, K., & Schreuder, R. (1998). Producing words in a foreign language: Can speakers prevent interference from their first language? *Bilingualism: Language and Cognition*, *1*(3), 213–229. 10.1017/s1366728998000364

[CR23] Kiyonaga, A., & Egner, T. (2014). The working memory Stroop effect: When internal representations clash with external stimuli. *Psychological Science,**25*(8), 1619–1629. 10.1177/0956797614536739 24958685 10.1177/0956797614536739 PMC4275427

[CR24] Li, P., Zhang, F., Yu, A., & Zhao, X. (2019). Language History Questionnaire (LHQ3): An enhanced tool for assessing multilingual experience. *Bilingualism: Language and Cognition*, *23*(5), 938–944. 10.1017/s1366728918001153

[CR25] Logan, G. D., & Zbrodoff, N. J. (1979). When it helps to be misled: Facilitative effects of increasing the frequency of conflicting stimuli in a Stroop-like task. *Memory & Cognition,**7*(3), 166–174. 10.3758/bf03197535

[CR26] Lupker, S. J., Brown, P., & Colombo, L. (1997). Strategic control in a naming task: Changing routes or changing deadlines? *Journal of Experimental Psychology: Learning, Memory, and Cognition,**23*(3), 570–590. 10.1037/0278-7393.23.3.570

[CR27] MacLeod, C. M. (1991). Half a century of research on the Stroop effect: An integrative review. *Psychological Bulletin,**109*(2), 163–203. 10.1037/0033-2909.109.2.163 2034749 10.1037/0033-2909.109.2.163

[CR28] Oberauer, K., & Lewandowsky, S. (2019). Addressing the theory crisis in psychology. *Psychonomic Bulletin & Review,**26*(5), 1596–1618. 10.3758/s13423-019-01645-2 31515732 10.3758/s13423-019-01645-2

[CR29] Paap, K. (2022). *The bilingual advantage in executive functioning hypothesis: How the debate provides insight into psychology’s replication crisis*. Routledge. 10.4324/9781003308027

[CR30] Paap, K., Myuz, H., Anders-Jefferson, R., Mason, L., & Zimiga, B. (2019). On the ambiguity regarding the relationship between sequential congruency effects, bilingual advantages in cognitive control, and the disengagement of attention. *AIMS Neuroscience,**6*(4), 282–298. 10.3934/neuroscience.2019.4.282 32341984 10.3934/Neuroscience.2019.4.282 PMC7179349

[CR31] Protopapas, A. (2007). Check Vocal: A program to facilitate checking the accuracy and response time of vocal responses from DMDX. *Behavior Research Methods,**39*(4), 859–862. 10.3758/bf03192979 18183901 10.3758/bf03192979

[CR32] R Core Team (2022). R: A language and environment for statistical computing. R Foundation for Statistical Computing, Vienna, Austria. https://www.R-project.org/

[CR33] Santesteban, M., & Schwieter, J. (2020). Lexical selection and competition in bilinguals. In R. Heredia & A. Cieślicka (Eds.), *Bilingual lexical ambiguity resolution* (pp. 126–156). Cambridge, UK: Cambridge University Press. 10.1017/9781316535967.007

[CR34] Spinelli, G., Krishna, K., Perry, J. R., & Lupker, S. J. (2020). Working memory load dissociates contingency learning and item-specific proportion-congruent effects. *Journal of Experimental Psychology: Learning, Memory, and Cognition,**46*(11), 2007–2033. 10.1037/xlm0000934 32658541 10.1037/xlm0000934

[CR35] Spinelli, G., & Lupker, S. J. (2023). Robust evidence for proactive conflict adaptation in the proportion-congruent paradigm. *Journal of Experimental Psychology: Learning, Memory, and Cognition,**49*(5), 675–700. 10.1037/xlm0001144 35787140 10.1037/xlm0001144

[CR36] Spinelli, G., Perry, J. R., & Lupker, S. J. (2019). Adaptation to conflict frequency without contingency and temporal learning: Evidence from the picture–word interference task. *Journal of Experimental Psychology: Human Perception and Performance,**45*(8), 995–1014. 10.1037/xhp0000656 31144859 10.1037/xhp0000656

[CR37] Stroop, J. R. (1935). Studies of interference in serial verbal reactions. *Journal of Experimental Psychology,**18*(6), 643–662. 10.1037/h0054651

[CR38] Sulpizio, S., Toti, M., Del Maschio, N., Costa, A., Fedeli, D., Job, R., & Abutalebi, J. (2019). Are you really cursing? Neural processing of taboo words in native and foreign language. *Brain and Language,**194*, 84–92. 10.1016/j.bandl.2019.05.003 31146214 10.1016/j.bandl.2019.05.003

[CR39] Titone, D. A., & Tiv, M. (2023). Rethinking multilingual experience through a Systems Framework of Bilingualism. *Bilingualism: Language and Cognition*, *26*(1), 1–16. 10.1017/s1366728921001127

[CR40] van Heuven, W. J. B., Mandera, P., Keuleers, E., & Brysbaert, M. (2014). Subtlex-UK: A new and improved word frequency database for British English. *Quarterly Journal of Experimental Psychology,**67*(6), 1176–1190. 10.1080/17470218.2013.850521 10.1080/17470218.2013.850521 24417251

