# Supplementary material for: Is adaptation involved in bilingual language production? A fresh look at the assumptions motivating potential bilingual-monolingual differences in adaptive control
Source: Psychon Bull Rev. 2024 May 7;31(6):2681–91. doi: 10.3758/s13423-024-02503-6 (PMC11680624; doi:10.3758/s13423-024-02503-6)
Supplement: Supplementary file 1 — Supplementary file1 (DOCX 956 KB) [file 13423_2024_2503_MOESM1_ESM.docx]

**Recruitment tools**

To recruit participants, the study was advertised in social media groups associated with, and classes offered at, the University of Milano-Bicocca, through the university’s participant pool, and within the experimenters’ social circles. Here, we report the description of the study’s pre-screening test on the university’s participant pool. The study was described with similar wording with the other recruitment tools.

Original (Italian): Lo scopo generale del presente studio è indagare come le persone che conoscono due lingue (come l'italiano e l'inglese) riescano a parlare nella lingua che intendono utilizzare, in particolare quando questa è una lingua per loro non dominante (in cui sono meno competenti, es. l'inglese) e devono gestire possibili influenze dall’altra lingua che conoscono, la lingua dominante (in cui sono più competenti, es. l'italiano). In questo prescreening, le presenteremo un breve test di inglese, da svolgere online, per valutare la sua idoneità al resto dello studio. Il test si compone di 25 domande e durerà 5 minuti. Non sono previsti crediti per questo test, ma, se idonea/o, riceverà un invito a partecipare al resto dello studio, per il quale riceverà 0.6 crediti.

Requisiti: Parlanti nativi di italiano, capacità visive e uditive normali o rese normali (es. con occhiali), età compresa tra i 18 e i 45 anni, conoscenza dell'inglese al livello stabilito dal test.

English translation: The general purpose of the present study is to examine how people who know two languages (such as Italian and English) manage to speak in the language they intend to use, particularly when that language is a non-dominant language for them (a language in which they are less proficient, e.g., English) and they need to handle potential influences from the other language they know, the dominant language (the language in which they are more proficient, e.g., Italian). In this pre-screening test, we will present you with a short English test, to be completed on-line, in order to determine your eligibility for the rest of the study. The test comprises 25 questions and will last 5 minutes. There are no credits associated with this test, however, if you are eligible, you will receive an invitation to participate in the rest of the study, for which you will receive 0.6 credits.

Requirements: Native Italian speakers, normal or corrected-to-normal hearing and vision (e.g., with glasses), 18 to 45 years old, knowledge of English at the level established by the test.

**Pilot study**

Prior to the study reported in the main text, a pilot study was run in order to select the most appropriate stimuli for the L2 picture naming task, that is, pictures which would be 1) relatively easy to name and 2) clearly classifiable as either cognate or noncognate.

Method

*Participants*

We hypothesized that a sample size of 25 participants would provide reliable enough norms for the stimulus selection process (see “Stimulus selection process” below). Participants were recruited from the same population and using the same procedures as in the main study (see the main text and “Recruitment tools” above). The requirements to participate were also the same as in the main study. Among these, participants were required to pass an English pre-screening test (see “Materials and procedure” below). Sixty-six participants completed the pre-screening test. Of these, 42 passed it and 25 (i.e., our target sample size) came to the lab to complete the study. Since there were no exclusion criteria (see “Data analysis” below), no participant was excluded. Of the 25 participants, 17 self-identified as female and 8 as male, with 21.84 years of age on average (*SD* = 3.20, range = 19–34); 21 reported knowing a third language beside Italian and English and 6 a fourth language, although their proficiency, immersion, and dominance in those languages (as calculated using Li et al.’s, 2019, formulas—see “Materials and procedure” below) were lower than those reported for Italian or English on average; all were born in Italy except 2 who came to live in Italy during childhood; and all resided in Italy. We report additional information on Italian (L1) and English (L2), the two languages involved in the study, in Table S1.

Table S1

*Characteristics of Italian (L1) and English (L2) for participants in the pilot study*

|  | Italian (L1) | | | English (L2) | | |
| --- | --- | --- | --- | --- | --- | --- |
| Characteristic | Mean | SD | Range | Mean | SD | Range |
| Proficiency | .97 | .06 | .79–1 | .83 | .07 | .71–1 |
| Immersion | .88 | .08 | .60–.96 | .71 | .07 | .53–.85 |
| Dominance | .64 | .05 | .51–.73 | .47 | .06 | .37–.58 |
| Pre-screening score |  |  |  | 22.12 | 2.13 | 18–25 |

*Note*. Proficiency, immersion, and dominance are aggregated scores ranging from 0 to 1 calculated using the formulas in the LHQ3 (with a couple of corrections explained in “Materials and procedure” below; note that because of those corrections, dominance for the final sample could not be calculated for Italian in 2 cases). The pre-screening score is the sum of correct responses to the 25 questions included in Cambridge’s online test for adult learners of English (see “Materials and procedure”).

*Materials and procedure*

*Pre-screening session*

Participants were pre-screened using the same test used for the main study, the Cambridge’s online test for adult learners of English (<https://www.cambridgeenglish.org/test-your-english/general-english/>; see Sulpizio et al., 2020). The test comprises 25 multiple-choice questions that evaluate English grammatical and conversational knowledge and estimate proficiency within the Common European Framework of Reference for Languages (CEFRL). To pass the test, participants were required to respond correctly to at least 18 questions (out of 25), corresponding to an estimated B2 CEFRL level. The original test was re-created using the jspsych (de Leeuw, 2015) Javascript library hosted on Cognition (<https://www.cognition.run/>) and participants completed it remotely on their own devices. To participate in the pre-screening test and the subsequent sessions, participants expressed their informed consent. The study was approved by the university’s Ethics Board (protocol RM-2021-445).

*Lab session*

Participants who passed the pre-screening test were invited to participate in the lab session, which comprised a language background questionnaire and an L2 picture naming task. Because of the length and the effort required by the session, in order to reduce fatigue, no other task was included. All instructions were given in Italian. The whole session took about 1.5 hours to complete.

*Language background questionnaire*. To assess participants’ language background, we used the same questionnaire used for the main study, the Language History Questionnaire 3.0 (LHQ3; Li et al., 2019). For a description of the LHQ3, see the main text. Here, we explain how we calculated proficiency, immersion, and dominance. These are scores ranging from 0 to 1 obtained by aggregating responses from multiple items in the LHQ3 using the formulas provided by Li et al. (2019).

Specifically, proficiency is an average of the self-rated proficiency in the four language skills (i.e., listening, speaking, reading, and writing), indicated by participants on a 1–7 Likert scale and converted to a 0–1 scale. In the rare case in which a participant failed to report their proficiency for one or more of the language skills for a given language, the average was based on the proficiency reported for the other skills.

Immersion is based, for one half, on the average of the difference between the participant’s age and the Age of Acquisition (AoA) for each language ability (divided by the participant’s age), and for the other half, on the years of use of the language (also divided by the participant’s age). The two halves, each of which produces a theoretical maximum value of 1, are summed and then multiplied by .5 in order for the result to be in the 0–1 range. However, some of our participants, particularly for Italian, reported a number of years of use for a language that exceeded the maximum possible number of years of use suggested by their age and/or their AoA responses for that language (e.g., a 19-year-old participant who reported a minimum AoA of 1 for the language skills but 19 years of use for that language instead of the maximum possible value of 18; or even, a 18-year-old who reported 21 years of use for a language). Although it is still possible to calculate immersion in those cases, the result may not be a reliable one because one of the two terms of the formula (i.e., the term in the second half) could exceed its maximum value. Therefore, in those (rare) cases (as well as when the participant failed to report the years of use for a language), we replaced the years-of-use response for the relevant language with the maximum possible number of years of use, i.e., the difference between the participant’s age and the minimum AoA response for that language (in the first example noted above, the corrected years of use would be 18).

Finally, dominance is based, for one half, on proficiency, and for the other half, on the self-reported hours spent daily using the language with each of the four language skills divided by a constant. Again, the two halves, each of which produces a theoretical maximum value of 1, are summed and then multiplied by 0.5 in order for the result to be in the 0–1 range. Following Li et al. (2019), we used 16 as the constant in the second half, representing the typical number of awake hours in a day. However, some of our participants in the main study reported using a language ability in a given language for more than 16 hours. Again, although it is still possible to calculate dominance in those cases, the result may not be a reliable one because one of the two terms of the formula (i.e., the term in the second half) could exceed its maximum value. Here, given the severity of some of the discrepancies (up to 38 hours reported using one of the language skills), we suspected that the affected participants (i.e., 6 participants for Italian and 1 participant for English in the final sample of the main study, 1 participant for Italian in the pilot study) might not have fully understood the relevant items, at least for the relevant language, and we decided not to calculate dominance for that language. Further, dominance could not be calculated for an additional participant in the pilot study because she did not list Italian in the language-use items.

*L2 picture naming task*. Of the 750 colored drawings in the MultiPic dataset (Duñabeitia et al., 2018), 540 pictures were selected. We conducted this initial screening process to exclude stimuli which were unlikely to be ultimately selected for the main study (e.g., stimuli for which the Multipic norms suggested little agreement among L1 speakers on the Italian or English name, or stimuli which were not clearly classifiable as either cognate or noncognate) and to make the length of the pilot study more manageable. Each trial began with a fixation symbol (+) presented for 250 ms followed by the picture presented for 10,000 ms or until response. All pictures were 300-pixel wide and 300-pixel high. Participants were instructed to name the picture in English (their L2) with the name that they thought was the most appropriate. They were told to speak clearly, without hesitations, and not to worry excessively about their Italian accent. There was no emphasis on speed. To advance to the next trial, participants were required to press the space bar, and every 90 trials, they were invited to take a break. This slow-pace procedure was set up so that participants would have sufficient time to produce a response to each stimulus and would not feel excessively fatigued by the task. Prior to the experiment, participants completed a practice session with five new pictures. The order of presentation of the stimuli was randomized. DMDX (Forster & Forster, 2003) was used to program the task.

*Online session*

At the end of the lab session, the experimenter explained the instructions of the similarity rating task described below to the participants and sent a link to that task to their personal e-mail addresses. They were asked to complete that task on their personal computer within a week. We chose to administer this task in a separate session because the study would have been exceedingly long otherwise. We chose to administer it remotely because, since this session did not involve recording of vocal responses, there was no strong reason to administer it in person. Further, administering it remotely made it easier for participants to complete the study within a week in their own time rather than setting up an appointment with the experimenter at a time that was not completely of their choosing.

The materials used in the similarity rating task were the same pictures (including the practice ones) used in the L2 picture naming. However, the pictures were presented for reference only. More importantly, for each picture, we produced an audio file with the picture’s modal names (i.e., the name for which there was more agreement) in (L1) English and (L1) Italian in the Multipic norms (Duñabeitia et al., 2018) using Text-To-Speech (TTS) Automate (<https://ttsautomate.com/>). We used Amazon Polly as the TTS provider and selected the voices “Amy” and “Carla”, a British English female voice and an Italian female voice, for English and Italian, respectively. All the audio files were manually inspected to ensure that the pronunciation was correct. For each picture, the audio files containing the English and Italian name were then merged into a single audio file using SoX (<https://sox.sourceforge.net/>) with a 500-ms silence in the middle.

At the beginning of the experiment, all materials were loaded in the browser’s memory. Next, participants were asked to complete the study on a laptop or desktop computer in a single session without distractions. In particular, they were asked to silence all potential sources of audio other than the experiment. They were also invited to use desktop speakers or to wear headphones or earphones, if they had them. The experiment then entered full-screen mode and the instructions of the task were presented (because the Safari browser does not support keyboard input when the browser is in full-screen mode, participants were told not to use that browser, and the experiment stopped if they did use it). All instructions were given in Italian. In the instructions, participants were told that each trial would involve a visual presentation of one of the pictures used in the L2 picture naming task and an auditory presentation of its typical English and Italian names, with the English name not necessarily being the one that they had used to respond to that picture in the naming task. Their task was to indicate how similar the two names were on the basis of their pronunciation only. They were told to ignore other aspects for which the two names could be judged to be similar (e.g., how the names were spelled and how closely their meaning corresponded). The response was to be indicated by positioning a slider on a horizontal bar whose left endpoint represented no similarity at all and the right endpoint represented complete similarity.

On each trial, both the picture and the associated audio file were presented simultaneously. Below the picture, the following question was diplayed: “Quanto sono simili i nomi in inglese e italiano di questa figura, considerando soltanto la loro pronuncia?” (“How similar are the English and Italian names of this picture, considering their pronunciation only?”). Below the question, a 500-pixel, grey horizontal bar was displayed. At the left and the right ends of the bar, the labels “Per niente” (“Not at all”) and “Del tutto” (“Completely”), respectively, were displayed. Below the bar, two buttons were displayed, one next to the other. The button on the left, reading “Riascolta” (“Listen again”), allowed participants to listen to the audio file again for as many times as they needed. The button on the right, reading “Conferma” (“Confirm”), allowed participants to confirm their response. The former button was disabled until the audio file finished playing. The latter button was disabled until participants clicked on a point on the bar, which made a rectangular slider appear in that position (before this click, the slider was set in the middle of the bar in invisible mode). Participants could adjust the slider’s position on the bar by dragging it to another position or clicking on another point on the bar. When participants pressed either button, the value of the position of the slider was recorded. That value was an integer ranging from 0 (corresponding to the leftmost position) to 100 (corresponding to the rightmost position; the default value, that is, the value when the slider was invisible, was 50). After participants confirmed their response, the next trial started. All stimuli within a trial were centered on the screen, with Open Sans 18-pt font being used for text. The trials were presented in six blocks, each containing about 90 trials (by mistake, some of the blocks contained slightly more trials, e.g., 91, or slightly fewer trials, e.g., 88, than 90). There was a self-paced pause between blocks. Prior to the experiment, participants completed a practice session with the same 5 practice pictures used in the L2 picture naming task. By mistake, 23 of the participants were also presented with one of the test pictures in the practice session rather than in the test session. The order of presentation of the stimuli was randomized. The jspsych (de Leeuw, 2015) Javascript library hosted on Cognition (<https://www.cognition.run/>) was used to program the task. The task took about 1.5 hours to complete.

*Data analysis*

For both the pilot study and the main study, pre-screening and questionnaire data were inspected for potential discrepancies, inconsistencies, and missing data. A detailed log of these cases and the actions taken to address them is included in the OSF repository.

The waveforms of responses to the L2 picture naming task were manually inspected with CheckVocal (Protopapas, 2007) to determine the correct placement of timing marks and to classify the response as belonging to one of 9 categories: 1) “exact”: The response was the modal name reported in the Multipic norms for English (henceforth, “modal name”) and was pronounced as it was expected (67.90% of the data); 2) “pronunciation error”: The response revealed that the participant was attempting to produce the modal name, but it contained one or more pronunciation errors, most typically affecting vowel phonemes (e.g., mountain being pronounced ['mɔntain] instead of [ˈmaʊntɪn], the correct pronunciation; 1.66% of the data); 3) “number error”: The response revealed that the participant was attempting to produce the modal name, but they used the plural when the singular was expected or (less frequently) vice versa (e.g., “veins” instead of “vein”; .68% of the data); 4) “abbreviation/expansion error”: The response involved the modal name, but it was either an abbreviated form (e.g. “mic” instead of “microphone”) or an expanded form (e.g., “bicycle” instead of “bike”) of the modal name (note that this case also included the use of fewer/more morphemes than in the modal name, e.g., “saw” instead of “chainsaw” and “water fountain” instead of “fountain”; 1.56% of the data); 5) “lexical error”: The response was an English word, but not the modal name (e.g., “rock” instead of “stone”; 22.70% of the data); 6) “other error”: The response was either an Italian word (e.g., “ananas” instead of “pineapple”), a mispronounced English word that was not the modal name (e.g., [berd], likely meaning “beard”, instead of “razor”) or that reflected the U.S. pronunciation of the modal name (e.g., “airplane” instead of “aeroplane”), an English-Italian false friend (e.g., “magazine”, similar to the Italian “magazzino”, instead of “warehouse”), a made-up English-Italian hybrid (e.g., [im'bjut], an anglicization of the Italian “imbuto”, instead of “funnel”), or a statement such as “I don’t know” (1.56% of the data); 7) “unintelligible”: The response was either incomplete, some sort of hesitation (e.g., “um”), or spoken too softly to be understood (.13% of the data); 8) “mistrigger”: The microphone was triggered by some noise that was not recognizable as the participant’s voice (e.g., a door slamming; .41% of the data); 9) “null”: There was no response (3.40% of the data). All responses were also transcribed using either their English spelling (for categories 1, 3, 4 and 5), their Italian spelling (for the Italian words in category 6), or the International Phonetic Alphabet (for categories 2 and 7). The subcases in categories 6 and 7 were also transcribed. The result of the categorization process was then used in the stimulus selection process described below.

The final response to each of the pictures in the similarity rating task—as noted above, an integer ranging from 0 (reflecting no similarity at all between the English and Italian names of the picture) to 100 (reflecting complete similarity)—were averaged across participants. These averages were also used in the stimulus selection process.

Stimulus selection process

Two constraints were used to select the pictures for the main study. First, in order to ensure that the pictures selected for the main study would be named with relative ease, we constrained the selection to pictures with a high enough proportion of “exact” responses in the L2 picture naming task. A histogram of the percentage of exact responses for the 540 pictures is presented in Figure S1A. We constrained the selection to the 294 pictures included in the three rightmost bars of the graph, that is, pictures which elicited a percentage of exact responses of at least 70%.

Second, we constrained the selection to pictures which would be clearly classifiable as either cognate or noncognate as suggested by participants’ similarity ratings. A histogram of the similarity ratings for the 540 pictures is presented in Figure S1B. Note that most of the ratings were concentrated in the leftmost bar of the graph; for the other ratings, the distribution was more even. Note further that stimuli with intermedium ratings involved quite a bit of similarity (e.g., with a rating of 50.52 was the pair formed by “soap” and “sapone”, which share two consonants). This fact suggests that, in the similarity rating task, relatively low values of the rating scale were associated with a fair degree of similarity. Ultimately, we constrained the selection of cognates to pictures associated with similarity ratings of at least 60 (164 pictures) and the selection of noncognates to pictures associated with ratings of at most 20 (312 pictures).

Applying both constraints, there were 106 eligible cognate pictures and 154 eligible noncognate pictures. From there, we tried to select as many cognate and noncognate pictures as possible, while matching the two types of stimuli on as many relevant characteristics as possible. Ultimately, we selected 96 cognate and 96 noncognate pictures matched on the percentage of exact responses in the pilot study (*M* = 89.22% and *M* = 88.66%, respectively), their visual complexity (*M* = 2.46 and *M* = 2.36, respectively, as per the Multipic norms), and the Zipf frequency of the exact response (*M* = 4.08 and *M* = 4.13, respectively, extracted from Subtlex-UK, van Heuven et al., 2014). We were not able to match a large enough number of cognate and noncognate pictures on other potentially relevant characteristics, particularly the number of syllables of the exact response, which was higher for cognate than for noncognate pictures (*M* = 2.18 and *M* = 1.50, respectively; see also Table 2 in the main text). The reason is that the exact responses for our cognate pictures were generally longer than those for noncognate pictures in our sample, likely a reflection of the fact that English words in an English-Italian cognate pair (typically words of Latin origin, e.g., “elephant” from Latin “elephantus”) tend to be longer than English words in an English-Italian noncognate pair (typically words of Germanic origin, e.g., “goat” from Old English “gat”; see, e.g., Bar-Ilan & Berman, 2007).

However, note that, first, this particular mismatch worked *against*, not *in favor of*, our manipulation. That is, to the extent that the length difference between the cognate and noncognate stimuli we selected could have had an impact in the main study, it would have been for a slowdown for cognate stimuli compared to noncognate stimuli, i.e., a reduction of the cognate effect. Second and most importantly, the aim of the main study was not so much to produce a completely confound-free cognate effect; rather, it was to examine whether the proportion of cognate and noncognate pictures in the list would have an impact on the size of the cognate effect. As explained in the main text, that potential impact is theoretically motivated by the idea that adaptive control is involved in bilingual language production. In contrast, there is no theoretical motivation to hypothesize such an impact for other variables that were correlated with cognate status in our study such as the number of syllables (i.e., there is no reason to believe that the naming difference between pictures with short vs. long names would be affected by the proportion of the two types of pictures in the list).

Figure S1

*Distribution of the percentage of exact responses and the average similarity rating, respectively, for the 540 pictures used in the pilot study*


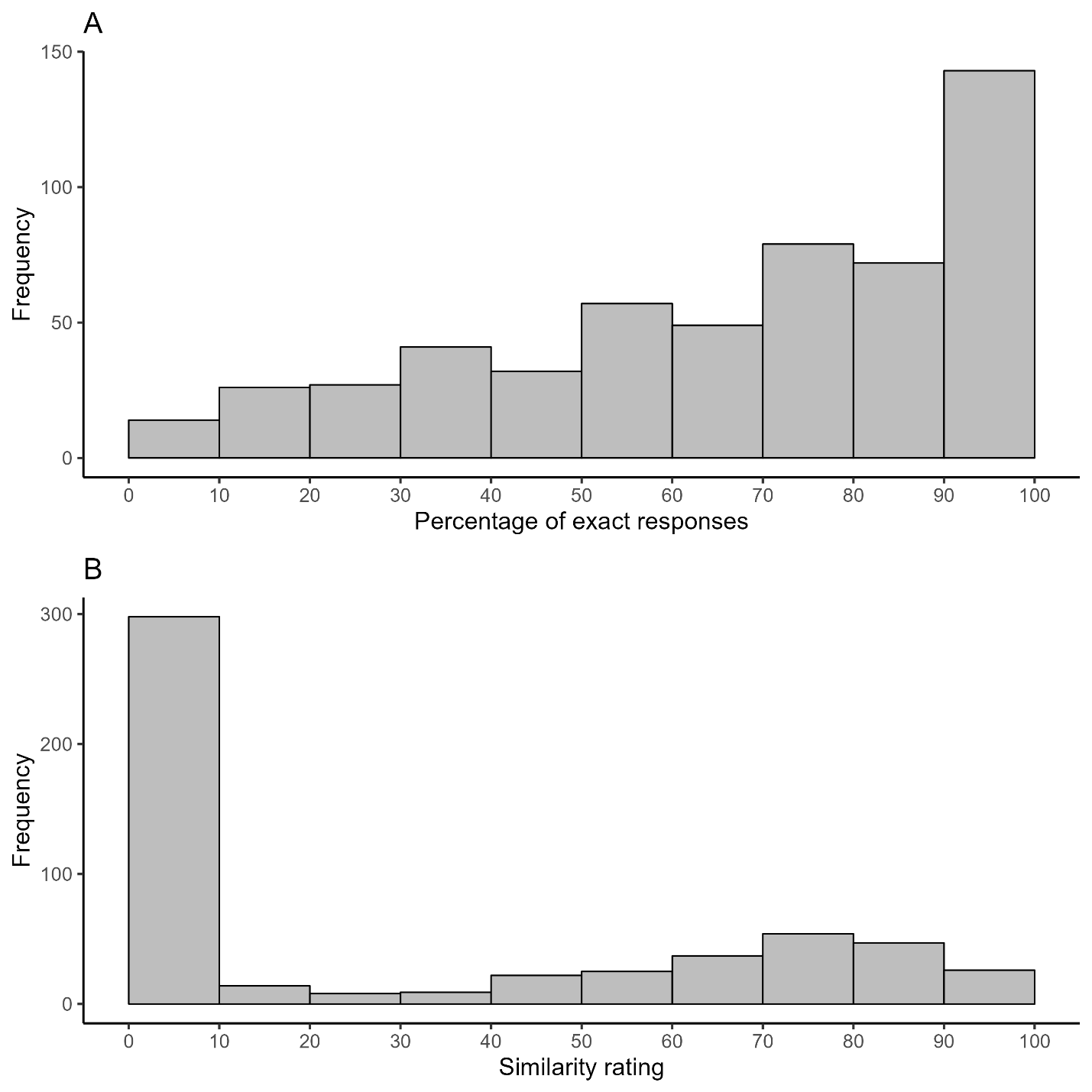


**Functions and packages used in mixed-effects analyses**

The lmer function in the *lmerTest* package, version 3.1-3 (Kuznetsova et al., 2017), and the glmer function in the *lme4* package, version 1.1-30 (Bates et al., 2015b), were used to fit the linear and generalized linear mixed-effects models, respectively, and obtain probability estimates. The emmeans function in the *emmeans* package, version 1.7.5 (Lenth, 2022), was used to conduct follow-up analyses. The lmBF function in the *BayesFactor* package, version 0.9.12.4.4 (Morey & Rouder, 2022), was used to fit RT models with and without the key interaction between List Type and Cognate Status (for L2 picture naming)/Congruency (for L1 Stroop).

**Additional analyses**

Best-fitting models

For both L2 picture naming and L1 Stroop, the step function in the lmerTest package was applied to the RT models reported in the main text in order to obtain the best-fitting model using backward selection. The full results are reported in Table S2. As noted in the main text, the best-fitting model for L2 picture naming did not include the interaction between Cognate Status and List Type, whereas the best-fitting model for L1 Stroop did not include the random effect of target color.

Table S2

*Variances and standard deviations for the random effects and coefficients, standard errors, statistics and probability values for the fixed effects used in the best-fitting models of response times in the L2 picture naming task and the L1 Stroop task*

| L2 picture naming | | | | | L1 Stroop | | | | |
| --- | --- | --- | --- | --- | --- | --- | --- | --- | --- |
| Random effects | *Variance* | *SD* |  |  | Random effects | *Variance* | *SD* |  |  |
| Participant (intercept) | 19770.09 | 140.61 |  |  | Participant (intercept) | 11415.09 | 106.84 |  |  |
| Picture (intercept) | 33684.44 | 183.53 |  |  |  |  |  |  |  |
| Fixed effects | *β* | *SE* | *t* | *p* | Fixed effects | *β* | *SE* | *t* | *p* |
| Intercept | 1225.48 | 24.56 | 49.89 | < .001 | Intercept | 741.47 | 15.49 | 47.85 | < .001 |
| Cognate Status | -39.36 | 14.02 | -2.81 | .005 | Congruency | -39.51 | 1.50 | -26.27 | < .001 |
| List Type | -8.99 | 4.55 | -1.97 | .048 | List Type | 5.57 | 1.50 | 3.71 | < .001 |
|  |  |  |  |  | Congruency × List Type | -8.49 | 1.50 | -5.64 | < .001 |

Full sample

For both L2 picture naming and L1 Stroop, we conducted the same analyses reported in the main text but with no participant excluded. That is, we used the full sample of 60 participants even though some performed poorly on the L2 picture naming task.

For the L2 picture naming task, the mean participant-based RTs and error rates are presented in Table S3 and the full results are reported in Table S4. The pattern of results was the same as with the sample used in the analyses reported in the main text, with the exception that there was a main effect of Cognate Status in the accuracy data (cognate more accurate than noncognate), an effect which was not significant in the main-text analysis. Most importantly, there was no interaction between Cognate Status and List Type in the RTs (or the error rates), and the Bayes Factor, *BF*_10_ = .05 ±4.8%, still favored the additive model over the interactive one.

Table S3

*Mean participant-based response times and percentage error rates (and corresponding 95% confidence intervals calculated using Cousineau’s (2019) method) in the L2 picture naming task using the full sample*

|  | Response times | | Error rates | |
| --- | --- | --- | --- | --- |
| Cognate status | Mostly-cognate list | Mostly-noncognate list | Mostly-cognate list | Mostly-noncognate list |
| Cognate | 1176 [1152, 1201] | 1194 [1169, 1219] | 13.39 [11.45, 15.33] | 12.84 [10.85, 14.82] |
| Noncognate | 1276 [1246, 1306] | 1286 [1259, 1314] | 16.72 [13.49, 19.95] | 17.20 [14.22, 20.19] |
| Cognate effect | 100 | 92 | 3.33 | 4.36 |

Table S4

*Variances and standard deviations for the random effects and coefficients, standard errors, statistics, and probability values for the fixed effects used in the models of response times and accuracy in the L2 picture naming task using the full sample*

|  | Response times | | | | Accuracy | | | |
| --- | --- | --- | --- | --- | --- | --- | --- | --- |
| Random effects | *Variance* | *SD* |  |  | *Variance* | *SD* |  |  |
| Participant (intercept) | 22625.74 | 150.42 |  |  | .632 | .795 |  |  |
| Picture (intercept) | 33950.11 | 184.26 |  |  | 1.342 | 1.159 |  |  |
| Fixed effects | *β* | *SE* | *t* | *p* | *β* | *SE* | *z* | *p* |
| Intercept | 1259.29 | 23.95 | 52.59 | < .001 | 2.275 | .140 | 16.24 | < .001 |
| Cognate Status | -42.64 | 13.99 | -3.05 | .003 | .234 | .093 | 2.52 | .012 |
| List Type | -9.35 | 4.30 | -2.18 | .030 | -.008 | .036 | -.22 | .826 |
| Cognate Status × List Type | -2.23 | 4.30 | -.52 | .604 | -.015 | .036 | -.43 | .669 |

*Note.* The accuracy coefficients are in the logit scale, not in the response scale.

For the L1 Stroop task, the mean participant-based RTs and error rates are presented in Table S5 and the full results are reported in Table S6. The pattern of results was the same as with the sample used in the analyses reported in the main text, with the exception that, in the RT model, there was no main effect of List Type, an effect which was significant in the main-text analysis. Most importantly, there was still an interaction between Congruency and List Type in the RTs, and the Bayes Factor, *BF*_10_ = 4961186 ± 3.07%, still favored the interactive model over the additive one.

Table S5

*Mean participant-based response times and percentage error rates (and corresponding 95% confidence intervals calculated using Cousineau’s (2019) method) in the L1 Stroop task using the full sample*

|  | Response times | | Error rates | |
| --- | --- | --- | --- | --- |
| Congruency | Mostly-congruent list | Mostly-incongruent list | Mostly-congruent list | Mostly-incongruent list |
| Congruent | 699 [683, 716] | 713 [698, 728] | .28 [.03, .53] | .28 [0, .56] |
| Incongruent | 803 [785, 821] | 783 [764, 801] | 3.25 [2.05, 4.45] | 2.07 [1.30, 2.83] |
| Congruency effect | 104 | 70 | 2.97 | 1.79 |

Table S6

*Variances and standard deviations for the random effects and coefficients, standard errors, statistics, and probability values for the fixed effects used in the models of response times and accuracy in the L1 Stroop task using the full sample*

|  | Response times | | | | Accuracy | | | |
| --- | --- | --- | --- | --- | --- | --- | --- | --- |
| Random effect | *Variance* | *SD* |  |  | *Variance* | *SD* |  |  |
| Participant (intercept) | 11232.30 | 105.98 |  |  | .330 | .575 |  |  |
| Color (intercept) | 4.05 | 2.01 |  |  | .003 | .052 |  |  |
| Fixed effect | *β* | *SE* | *t* | *p* | *β* | *SE* | *z* | *p* |
| Intercept | 749.14 | 13.79 | 54.33 | < .001 | 4.915 | .160 | 30.67 | < .001 |
| Congruency | -43.01 | 1.39 | -30.99 | < .001 | 1.130 | .130 | 8.71 | < .001 |
| List Type | 1.36 | 1.39 | .98 | .327 | -.117 | .130 | -.90 | .368 |
| Congruency × List Type | -8.54 | 1.39 | -6.15 | < .001 | .119 | .130 | .92 | .358 |

*Note.* The accuracy coefficients are in the logit scale, not in the response scale.

Maximal random structure allowed by the data

For both L2 picture naming and L1 Stroop, we conducted the same analyses reported in the main text but specifying the maximal random structure allowed by the data (Bates et al., 2015a). That is, we first ran a model with the appropriate maximal random structure for our design (Barr et al., 2013). For L2 picture naming, the appropriate maximal random structure for our design included, for participants, the random intercept, the random slope of Cognate Status, List Type, and their interaction, and the correlations between these parameters; for pictures (i.e., the target stimuli in that experiment), the structure included the random intercept, the random slope of List Type, and their correlation. For L1 Stroop, the appropriate maximal random structure for our design included, for both participants and colors (i.e., the target stimuli in that experiment), the random intercept, the random slope of Congruency, List Type, and their interaction, and the correlations between these parameters. Next, in case the model with the maximal random structure did not converge, we reduced the random structure using the procedure recommended by Bates et al. (2015a) until the model converged successfully.

For the L2 picture naming task, the full results are reported in Table S7. For RTs, the maximal random structure allowed by the data included, for participants, the random intercept and the random slopes of Cognate Status and List Type, and for pictures, the random intercept, the random slope of List Type, and their correlation. For accuracy, the maximal random structure allowed by the data included, for participants, the random intercept and the random slope of Cognate Status, and for pictures, the random intercept. The pattern of results for the fixed effects was the same as with the sample used in the analyses reported in the main text: In the RTs, there was a main effect of Cognate Status, the main effect of List Type fell short of significance, and there was no indication of there being an interaction; in the error rates, no effect was significant. Also similar to the analyses conducted without the maximal random structure, applying the step function in the lmerTest package to the RT model reported in Table S7 produced a best-fitting model, reported in Table S9, including no interaction and in which both of the main effects were significant. The random structure was also reduced, including, for participants, the random intercept and the random slope of Cognate Status and, for pictures, the random intercept.

For the L1 Stroop task, the full results are reported in Table S8. For RTs, the maximal random structure allowed by the data included the random intercept for colors and, for participants, the random intercept, the random slopes of Cognate Status and List Type, their interaction, and the correlation between these parameters. For accuracy, the maximal random structure allowed by the data included the random intercept and the random slope of Congruency for participants. The pattern of results for the fixed effects was the same as in the analyses reported in the main text, with the exception that, in the RT model, there was no main effect of List Type, an effect which was significant in the main-text analysis. The best-fitting RT model, a model in which the random intercept for colors was dropped, produced nearly identical results (see Table S9; note that this model failed to converge initially, but did converge after being restarted from the apparent optimum as suggested by lme4’s troubleshooting procedure—see ‘convergence’ help page in R).

Table S7

*Variances, standard deviations, and correlations for the random effects and coefficients, standard errors, statistics, and probability values for the fixed effects used in the models of response times and accuracy in the L2 picture naming task using the maximal random structure allowed by the data*

|  | Response times | | | | Accuracy | | | |
| --- | --- | --- | --- | --- | --- | --- | --- | --- |
| Random effects | *Variance* | *SD* | *Correlation* |  | *Variance* | *SD* |  |  |
| Participant (intercept) | 19865.22 | 140.94 |  |  | .266 | .516 |  |  |
| Cognate Status | 583.24 | 24.15 |  |  | .002 | .046 |  |  |
| List Type | 63.62 | 7.98 |  |  |  |  |  |  |
| Picture (intercept) | 33708.01 | 183.60 |  |  | 1.358 | 1.165 |  |  |
| List Type | 47.09 | 6.86 | .121 |  |  |  |  |  |
| Fixed effects | *β* | *SE* | *t* | *p* | *β* | *SE* | *z* | *p* |
| Intercept | 1227.48 | 24.71 | 49.67 | < .001 | 2.547 | .125 | 20.32 | < .001 |
| Cognate Status | -39.95 | 14.45 | -2.76 | .006 | .116 | .097 | 1.19 | .234 |
| List Type | -8.89 | 4.72 | -1.89 | .065 | .026 | .041 | .64 | .525 |
| Cognate Status × List Type | -3.26 | 4.57 | -.71 | .477 | -.003 | .041 | -.07 | .946 |

*Note.* The accuracy coefficients are in the logit scale, not in the response scale.

Table S8

*Variances, standard deviations, and correlations for the random effects and coefficients, standard errors, statistics, and probability values for the fixed effects used in the models of response times and accuracy in the L1 Stroop task using the maximal random structure allowed by the data*

|  | Response times | | | |  | Accuracy | | | |
| --- | --- | --- | --- | --- | --- | --- | --- | --- | --- |
| Random effects | *Variance* | *SD* | *Correlation* | | | *Variance* | *SD* |  |  |
| Participant (intercept) | 11527.15 | 107.36 |  |  |  | .148 | .385 |  |  |
| Congruency | 680.83 | 26.09 | -.386 |  |  | .237 | .487 |  |  |
| List Type | 606.46 | 24.63 | .177 | .217 |  |  |  |  |  |
| Congruency × List Type | 47.33 | 6.88 | .505 | .360 | .271 |  |  |  |  |
| Color (intercept) | 7.42 | 2.72 |  |  |  |  |  |  |  |
| Fixed effects | *β* | *SE* | *t* | *p* |  | *β* | *SE* | *z* | *p* |
| Intercept | 741.79 | 15.62 | 47.48 | < .001 |  | 4.893 | .166 | 29.41 | < .001 |
| Congruency | -39.79 | 4.04 | -9.86 | < .001 |  | 1.073 | .160 | 6.69 | < .001 |
| List Type | 5.60 | 3.84 | 1.46 | .151 |  | -.117 | .142 | -.83 | .409 |
| Congruency × List Type | -8.46 | 1.76 | -4.80 | < .001 |  | .118 | .142 | .83 | .405 |

*Note.* The accuracy coefficients are in the logit scale, not in the response scale.

Table S9

*Variances, standard deviations, and correlations for the random effects and coefficients, standard errors, statistics, and probability values for the fixed effects used in the best-fitting models of response times in the L2 picture naming task and the L1 Stroop task initially involving the maximal random structure allowed by the data*

| L2 picture naming | | | | | L1 Stroop | | | | |  |
| --- | --- | --- | --- | --- | --- | --- | --- | --- | --- | --- |
| Random effects | *Variance* | *SD* |  |  | Random effects | *Variance* | *SD* | *Correlation* | | |
| Participant (intercept) | 19869.54 | 140.96 |  |  | Participant (intercept) | 11523.69 | 107.35 |  |  |  |
| Cognate Status | 593.55 | 24.36 |  |  | Congruency | 680.97 | 26.10 | -.386 |  |  |
| Picture (intercept) | 33763.86 | 183.75 |  |  | List Type | 606.48 | 24.63 | .176 | .218 |  |
|  |  |  |  |  | Congruency × List Type | 47.30 | 6.88 | .505 | .360 | .271 |
| Fixed effects | *β* | *SE* | *t* | *p* | Fixed effects | *β* | *SE* | *t* | *p* |  |
| Intercept | 1225.86 | 24.61 | 49.80 | < .001 | Intercept | 741.79 | 15.56 | 47.66 | < .001 |  |
| Cognate Status | -39.89 | 14.47 | -2.76 | .006 | Congruency | -39.79 | 4.04 | -9.85 | < .001 |  |
| List Type | -9.07 | 4.54 | -2.00 | .046 | List Type | 5.60 | 3.84 | 1.46 | .151 |  |
|  |  |  |  |  | Congruency × List Type | -8.46 | 1.76 | -4.80 | < .001 |  |

Additional predictors

We conducted three sets of analyses with additional predictors for exploratory purposes. In the first set, conducted for both L2 picture naming and L1 Stroop, the additional predictors were language-based measures from the LHQ3 and the L1-to-L2 translation task. In the second set, conducted for L2 picture naming only, the additional predictors were performance-based measures from the L1 Stroop task. The reason these two analyses in particular are exploratory is that the present study was not set up to examine the impact of individual differences in language- or performance-based abilities, as we made no attempt to produce variability in those abilities in our sample, nor was our sample of the typical size used in individual-differences research. That said, these analyses might provide valuable insights, as explained below. Finally, in the third set of analyses, conducted for both L2 picture naming and L1 Stroop, the additional predictor was the the order in which the two list types in each task were administered (i.e., mostly-cognate first vs. mostly-noncognate first for L2 picture naming and mostly-congruent first vs. mostly-incongruent first for L1 Stroop).

*Language-based predictors*

We focused on L2 measures because of their relevance to L2 picture naming and because there was little variability in L1 measures. In particular, we focused on the aggregated scores produced by the LHQ3 (i.e., proficiency, immersion, and dominance) for English (i.e., the participants’ L2) and on English lexical fluency as measured by our L1-to-L2 translation task. To avoid multicollinearity issues, we inspected the pattern of correlation between these four variables. They tended to be positively correlated with one another, with the correlation between proficiency and dominance being particularly strong (see Table S10). Note, however, that this result was to be expected because, as explained above, proficiency is one of the variables making up the dominance score. More interesting were the moderate correlations between lexical fluency on one hand and proficiency and dominance on the other hand, as well as the fact that immersion was more weakly (and non-significantly) associated with the other three variables. Based on these results, we decided to use lexical fluency and immersion as the language-based predictors in our analyses. The reason we picked lexical fluency over proficiency and dominance is that, being an objective measure of participants’ L2 ability, lexical fluency is likely less subjected to bias than the other two measures, which are based on self-ratings (Tomoschuk et al., 2019; and note that, as discussed in “Materials and procedure” above, the issues with calculating dominance for some of the participants suggest that participants might not have always fully understood the relevant questionnaire items).

Table S10

*Pearson’s correlations between proficiency, immersion, dominance, and lexical fluency in English (L2)*

| Variables | 1. | 2. | 3. | 4. |
| --- | --- | --- | --- | --- |
| 1. L2 proficiency | 1 |  |  |  |
| 2. L2 immersion | .225 | 1 |  |  |
| 3. L2 dominance | .921*** | .102 | 1 |  |
| 4. L2 lexical fluency | .515*** | .266 | .492*** | 1 |

*Note*. *** *p* < .001; ** *p* <.01; * *p* < .05.

A reasonable prediction for L2 picture naming would seem to be that individuals with lower L2 immersion and/or lexical fluency should be those most likely to show a PC-like (i.e., interactive) pattern because they should produce a relatively large cognate effect (Costa et al., 2000) and should therefore have more to gain from adapting to the frequency of cognate and noncognate pictures in a list. This prediction is consistent with the controlled-dose hypothesis (Paap, 2018), the idea according to which individuals in earlier stages of learning a second language would be more likely to apply domain-general processes when using that language than more experienced bilinguals, individuals who would be more likely to have developed language-specific processes.

Concerning L1 Stroop, hypotheses of language-control associations (e.g., Bialystok, 2017) would seem to predict that lower L2 immersion and/or lexical fluency should be associated with some kind of performance benefit in the Stroop task, for example, faster RTs, lower error rates, and/or reduced congruency effects (e.g., Spinelli et al., 2022). Do note, however, that the verbal nature of the stimuli involved in a classic color-word Stroop task such as ours is not ideal to test those hypotheses, which have most typically been tested using non-linguistic tasks (e.g., Hilchey & Klein, 2011; Hilchey et al., 2015; Paap & Greenberg, 2013).

Both L2 Immersion and L2 Lexical Fluency were included in the RT and accuracy models with their main effects and their interactions with the original predictors (i.e., for L2 picture naming, Cognate Status and List Type; for L1 Stroop, Congruency and List Type). Interactions involving both Immersion and Lexical Fluency were excluded in order to reduce unnecessary model complexity. Both Immersion and Lexical Fluency were standardized before the analyses in order to help model convergence. The analyses were otherwise conducted as in the main text.

For L2 picture naming, the full results are reported in Table S11 (note that, in this case as well, the accuracy model failed to converge initially, but did converge after being restarted from the apparent optimum as per lme4’s troubleshooting procedure). The RT model showed, in addition to the main effects of Cognate Status and List Type previously discussed, a main effect of L2 Lexical Fluency, reflecting faster responses with higher fluency scores overall, and an interaction between Cognate Status and L2 Lexical Fluency. Follow-up analyses revealed that the interaction reflected the fact that, although both cognate and noncognate pictures were responded to faster with higher fluency scores (*β* = -53.2, *SE* = 19.8, *z* = -2.69, *p* = .007, and *β* = -88.8, *SE* = 19.8, *z* = -4.48, *p* < .001, respectively), this speed-up was more pronounced for the latter than for the former, leading to a reduction of the cognate effect with higher fluency scores (see Figure S2).

The accuracy model also showed a main effect of L2 Lexical Fluency, reflecting more accurate responses with higher fluency scores. However, this model also showed a main effect of L2 Immersion, reflecting more accurate responses with higher immersion scores overall, and two interactions which also involved L2 Immersion: the interaction between L2 Immersion and Cognate Status, reflecting the fact that L2 immersion mainly improved accuracy for noncognate pictures (*β* = .253, *SE* = .078, *z* = 3.24, *p* = .001; for cognate pictures, *β* = .073, *SE* = .080, *z* = .91, *p* = .362; see Figure S3A); and the interaction between L2 Immersion and List Type, reflecting the fact that L2 Immersion mainly improved accuracy in the mostly-cognate list (*β* = .259, *SE* = .078, *z* = 3.29, *p* = .001; for the mostly-noncognate list, *β* = .067, *SE* = .080, *z* = .84, *p* = .399; see Figure S3B).

Importantly, neither model showed a three-way interaction. Therefore, although L2 Immersion and L2 Lexical Fluency clearly had a (facilitatory) impact on L2 picture naming performance (with immersion mainly doing so for accuracy and fluency mainly doing so for RTs), this impact was orthogonal to our key manipulation involving the proportion of cognate and noncognate pictures in a list.

Table S11

*Variances and standard deviations for the random effects and coefficients, standard errors, statistics, and probability values for the fixed effects used in the models of response times and accuracy in the L2 picture naming task adding the language-based predictors*

|  | Response times | | | | Accuracy | | | |
| --- | --- | --- | --- | --- | --- | --- | --- | --- |
| Random effects | *Variance* | *SD* |  |  | *Variance* | *SD* |  |  |
| Participant (intercept) | 15359.23 | 123.93 |  |  | .125 | .354 |  |  |
| Picture (intercept) | 33733.06 | 183.67 |  |  | 1.360 | 1.166 |  |  |
| Fixed effects | *β* | *SE* | *t* | *p* | *β* | *SE* | *z* | *p* |
| Intercept | 1223.34 | 22.76 | 53.74 | < .001 | 2.560 | .113 | 22.57 | < .001 |
| Cognate Status | -39.41 | 14.03 | -2.81 | .005 | .106 | .098 | 1.09 | .277 |
| List Type | -9.04 | 4.55 | -1.99 | .047 | .040 | .043 | .94 | .345 |
| L2 Immersion | 14.86 | 18.89 | .79 | .436 | .163 | .068 | 2.41 | .016 |
| L2 Lexical Fluency | -70.98 | 19.22 | -3.69 | < .001 | .285 | .070 | 4.05 | < .001 |
| Cognate Status × List Type | -3.26 | 4.55 | -.72 | .474 | -.007 | .043 | -.18 | .861 |
| Cognate Status × L2 Immersion | -6.77 | 4.74 | -1.43 | .153 | -.090 | .041 | -2.18 | .029 |
| Cognate Status × L2 Lexical Fluency | 17.79 | 4.73 | 3.76 | < .001 | .035 | .045 | .76 | .446 |
| List Type × L2 Immersion | -2.15 | 4.78 | -.45 | .652 | .096 | .041 | 2.31 | .021 |
| List Type × L2 Lexical Fluency | -7.50 | 4.73 | -1.58 | .113 | -.019 | .046 | -.42 | .676 |
| Cognate Status × List Type × L2 Immersion | -.06 | 4.78 | -.01 | .990 | -.061 | .041 | -1.46 | .144 |
| Cognate Status × List Type × L2 Lexical Fluency | -5.27 | 4.74 | -1.11 | .266 | .049 | .046 | 1.08 | .278 |

*Note.* The accuracy coefficients are in the logit scale, not in the response scale.

Figure S2

*The impact of the L2 lexical fluency on RTs for cognate and noncognate pictures in the L2 picture naming task*


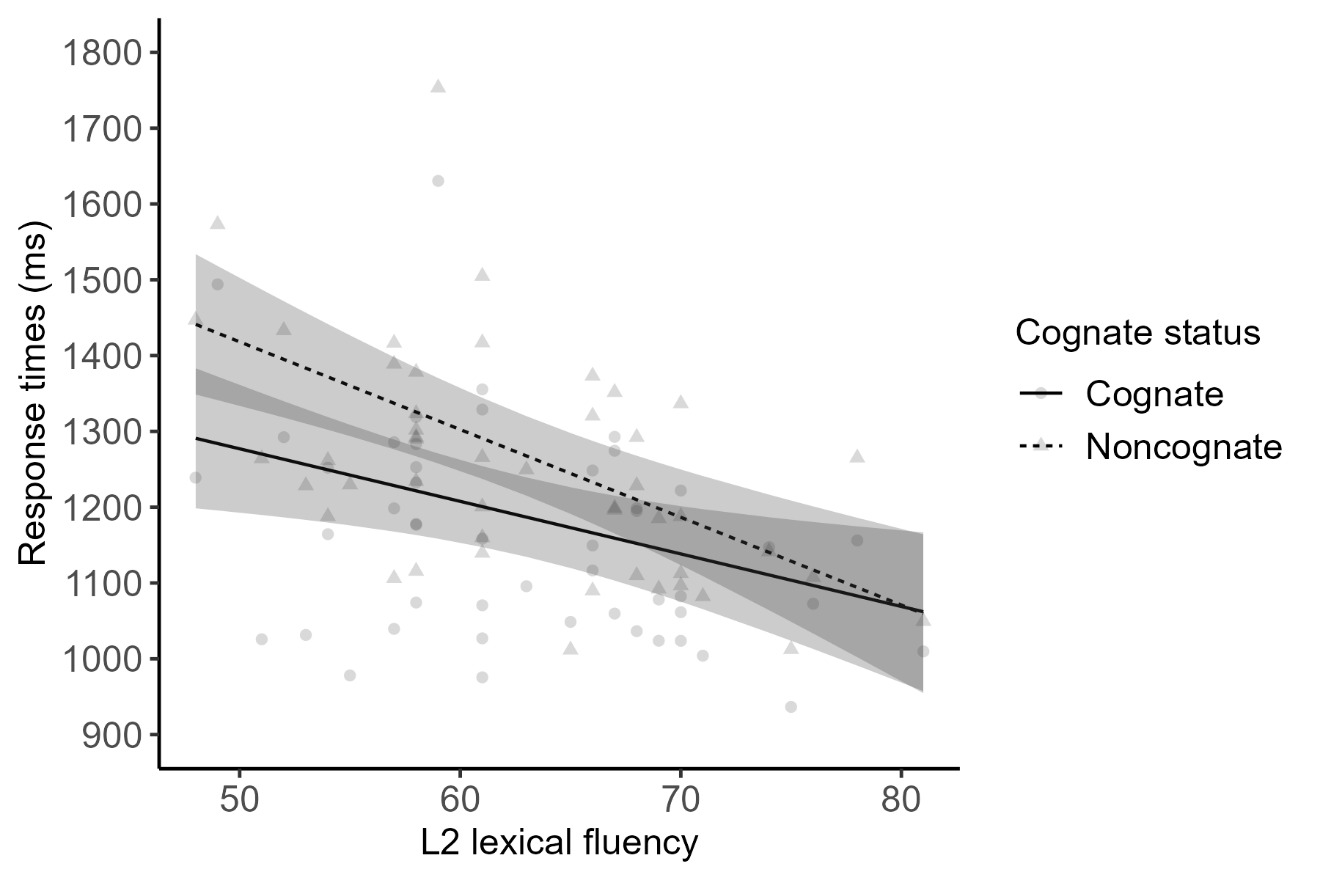


*Note.* The circles and triangles represent mean participant RTs and the lines (with 95% confidence bands) represent the model-estimated trends for cognate and noncognate pictures.

Figure S3

*The impact of the L2 immersion on error rates for cognate and noncognate pictures (A) and for mostly-cognate and mostly-noncognate lists (B) in the L2 picture naming task*

*
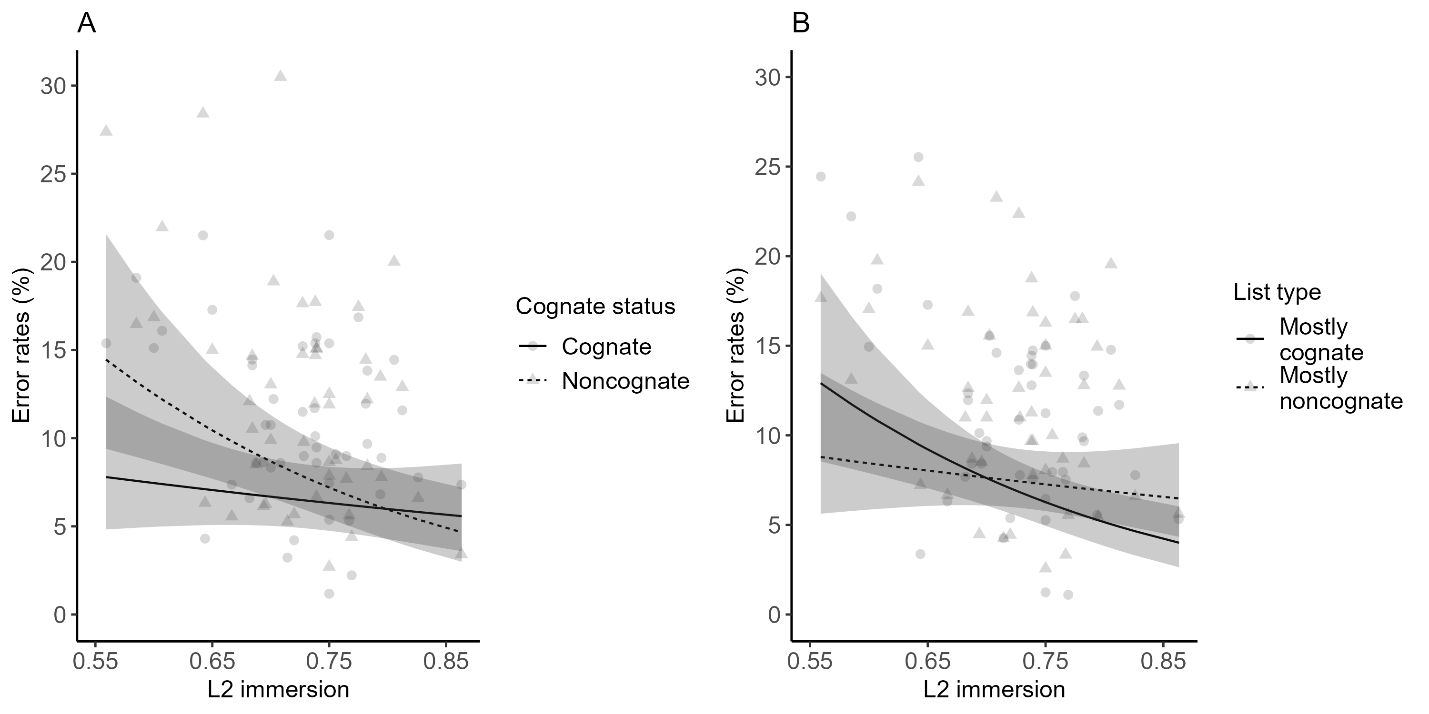
*

*Note.* The circles and triangles represent mean participant error rates and the lines (with 95% confidence bands) represent the model-estimated trends for cognate and noncognate pictures (A) or for mostly-cognate and mostly-noncognate lists (B). The reason the lines can show a curvature is that they were back-transformed from the logit scale used in the model to the response scale.

For L1 Stroop, the full results are reported in Table S12 (once again, the accuracy model failed to converge initially, but did converge after being restarted from the apparent optimum as per lme4’s troubleshooting procedure; note, further, that we did not include the random intercept for colors in that model because the previous analyses suggested that there was little variance associated with that intercept in our data). The RT model showed, in addition to the main effects of Congruency and List Type and the interaction between the two previously discussed, an interaction between List Type and L2 Immersion and an interaction between List Type and L2 Lexical Fluency. Follow-up analyses revealed that the former interaction reflected the fact that L2 Immersion was associated with faster RTs to a larger degree in the mostly-incongruent list than in the mostly-congruent list, although this speed-up was not significant in either list (*β* = -15.82, *SE* = 16.4, *z* = -.96, *p* = .335, and *β* = -3.09, *SE* = 16.4, *z* = -.19, *p* = .851, respectively; see Figure S4A); similarly, the latter interaction reflected the fact that L2 Lexical Fluency was associated, in this case, with *slower* RTs to a larger degree in the mostly-incongruent list than in the mostly-congruent list, although this slow-down was not significant in either list (*β* = 16.40, *SE* = 16.4, *z* = 1.00, *p* = .318, and *β* = 5.17, *SE* = 16.4, *z* = .31, *p* = .753, respectively; see Figure S4B).

The accuracy model only showed the main effect of Congruency. Overall, the language-based predictors seemed to have no clear impact on L1 Stroop performance (for a similar pattern of results, see Spinelli et al., 2022).

Table S12

*Variances and standard deviations for the random effects and coefficients, standard errors, statistics, and probability values for the fixed effects used in the models of response times and accuracy in the L1 Stroop task adding the language-based predictors*

|  | Response times | | | | Accuracy | | | |
| --- | --- | --- | --- | --- | --- | --- | --- | --- |
| Random effects | *Variance* | *SD* |  |  | *Variance* | *SD* |  |  |
| Participant (intercept) | 11765.15 | 108.47 |  |  | .287 | .536 |  |  |
| Color (intercept) | 6.68 | 2.59 |  |  |  |  |  |  |
| Fixed effects | *β* | *SE* | *t* | *p* | *β* | *SE* | *z* | *p* |
| Intercept | 741.49 | 15.78 | 46.99 | < .001 | 4.888 | .174 | 28.17 | < .001 |
| Congruency | -39.51 | 1.50 | -26.30 | < .001 | 1.092 | .146 | 7.50 | < .001 |
| List Type | 5.58 | 1.50 | 3.71 | < .001 | -.105 | .146 | -.72 | .469 |
| L2 Immersion | -9.45 | 16.34 | -.58 | .566 | .014 | .170 | .08 | .934 |
| L2 Lexical Fluency | 10.79 | 16.36 | .66 | .513 | .090 | .179 | .50 | .615 |
| Congruency × List Type | -8.50 | 1.50 | -5.66 | < .001 | .141 | .146 | .97 | .334 |
| Congruency × L2 Immersion | 1.18 | 1.56 | .75 | .451 | .158 | .149 | 1.06 | .288 |
| Congruency × L2 Lexical Fluency | -.14 | 1.56 | -.09 | .927 | - .138 | .159 | -.87 | .383 |
| List Type × L2 Immersion | 6.36 | 1.56 | 4.08 | < .001 | .114 | .149 | .76 | .445 |
| List Type × L2 Lexical Fluency | -5.62 | 1.56 | -3.60 | < .001 | .035 | .158 | .22 | .824 |
| Congruency × List Type × L2 Immersion | -.71 | 1.56 | -.45 | .650 | .076 | .149 | .51 | .608 |
| Congruency × List Type × L2 Lexical Fluency | 2.17 | 1.56 | 1.40 | .163 | .088 | .158 | .56 | .578 |

*Note.* The accuracy coefficients are in the logit scale, not in the response scale.

Figure S4

*The impact of the L2 immersion (A) and lexical fluency (B) on RTs for mostly-congruent and mostly-incongruent lists in the L1 Stroop task*

*
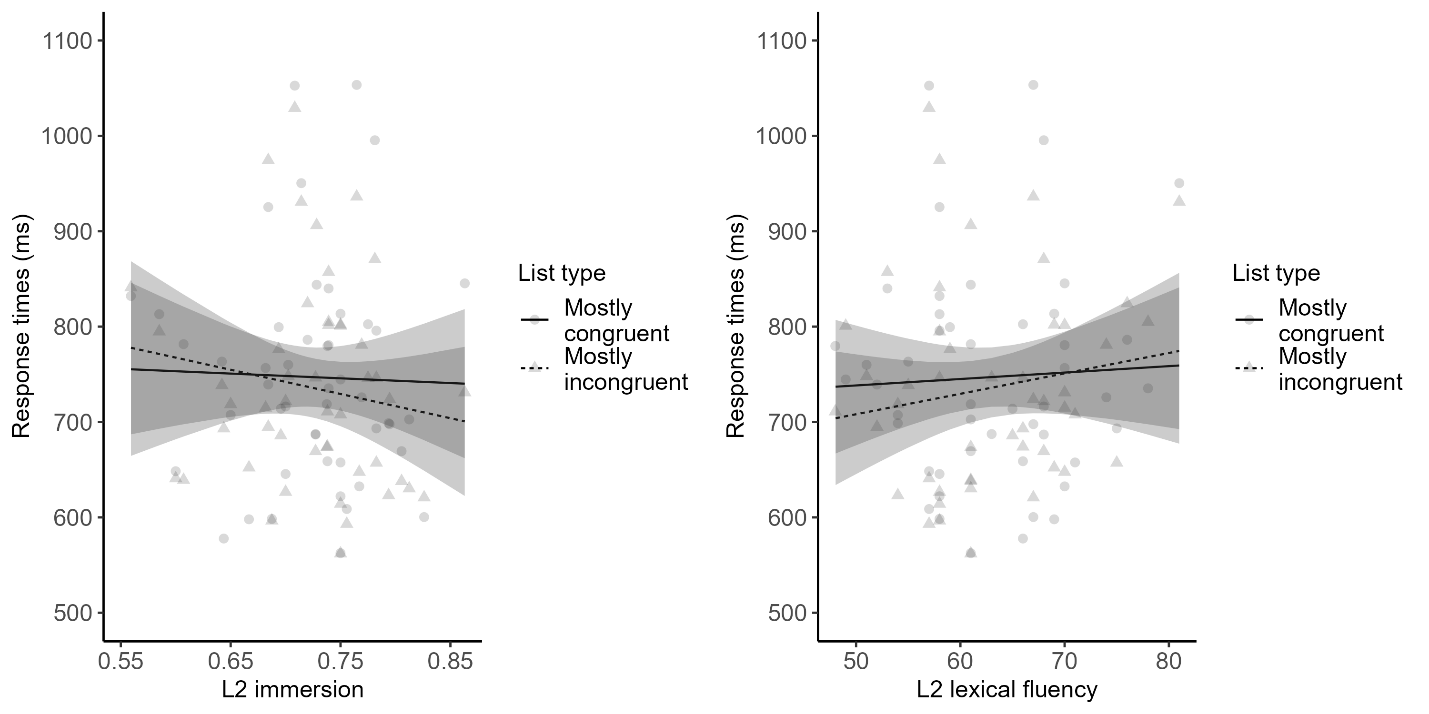
Note.* The circles and triangles represent mean participant RTs and the lines (with 95% confidence bands) represent the model-estimated trends for mostly-congruent and mostly-incongruent lists.

*Performance-based predictors*

With this analysis, we aimed to explore potential associations between performance measures on L2 picture naming and L1 Stroop using mixed-effects modelling as done so far. Because this type of analysis is similar to correlating performance across the two tasks, we deemed sufficient to report the analysis of only one of the tasks (i.e., L2 picture naming) as a function of performance in the other task (i.e., L1 Stroop) rather than presenting the reverse analysis (i.e., L1 Stroop performance as a function of L2 picture naming performance) as well. Further, we focused on RTs because it is in RTs that the key patterns emerged more clearly in both tasks.

The additional predictors we used were the participant’s mean congruency and PC effect in the L1 Stroop task. The former is defined as the difference between RTs for incongruent and congruent stimuli across both list types, and can be interpreted as an index of the participant’s inability to process task-relevant information selectively (i.e., the larger their congruency effect overall, the worse their processing selectivity). The latter is defined as the difference between the congruency effect in the mostly-congruent list and the congruency effect in the mostly-incongruent list, and can be interpreted as an index of the participant’s ability to adapt attention based on contextual information (i.e., the larger the PC effect, the better their adaptability; see Spinelli et al., 2022).

As with the language-based predictors, the L1 Stroop Congruency and PC effects were included in the RT model of L2 picture naming with their main effects and their interactions with the original L2 picture naming predictors, i.e., Cognate Status and List Type. In this case as well, both the congruency and the PC effect were standardized before the analyses to help model convergence. The analysis was otherwise conducted as in the main text.

The full results are reported on the left side of Table S13. In addition to the main effects of Cognate Status and List Type previously discussed, the results also showed an interaction between Cognate Status and L1 Stroop Congruency. Follow-up analyses revealed that the interaction reflected the fact that larger Stroop congruency effects were associated with faster RTs to a larger degree for noncognate pictures than for cognate pictures, although this speed-up was not significant for either type of picture (*β* = -34.6, *SE* = 21.8, *z* = -1.59, *p* = .112, and *β* = -15.5, *SE* = 21.8, *z* = -.71, *p* = .476, respectively). The result was a reduced cognate effect with larger Stroop congruency effects (see Figure S5A). In other words, cognate effects in L2 picture naming and congruency effects in L1 Stroop were negatively correlated.

To ensure that this pattern had to do with congruency effects and not with participants’ mean RTs in the Stroop task (as mean RTs and mean congruency effects in RTs in the Stroop task were positively correlated in our sample, *r* = .348, *p* = .015), we ran another analysis in which each participant’s L1 Stroop congruency and PC effects to be used as predictors of L2 picture naming performance were calculated after transforming that participant’s RTs in L1 Stroop into z-RTs, i.e., standardizing them. This procedure eliminated the correlation between mean overall z-RTs and mean congruency effects in z-RTs in the Stroop task, *r* = -.065, *p* = .660.

The full results of the analysis with L1 Stroop congruency and PC effects expressed in z-RTs are reported on the right side of Table S13. The pattern of results was the same as with L1 Stroop congruency and PC effects expressed in raw RTs. In particular, it was still the case that larger Stroop congruency effects were associated with faster RTs to a larger degree for noncognate pictures than for cognate pictures, a speed-up that, in this case, was significant for the former but not the latter type of picture (*β* = -49.3, *SE* = 21.1, *z* = -2.34, *p* = .020, and *β* = -30.0, *SE* = 21.1, *z* = -1.42, *p* = .155, respectively). As a result, cognate effects and Stroop congruency effects were negatively correlated (see Figure S5B).

Table S13

*Variances and standard deviations for the random effects and coefficients, standard errors, statistics, and probability values for the fixed effects used in the models of response times and accuracy in the L2 picture naming task adding the L1 performance predictors*

|  | With RT-based Stroop predictors | | | | With z-RT-based Stroop predictors | | | |
| --- | --- | --- | --- | --- | --- | --- | --- | --- |
| Random effects | *Variance* | *SD* |  |  | *Variance* | *SD* |  |  |
| Participant (intercept) | 19765.60 | 140.59 |  |  | 18545.95 | 136.18 |  |  |
| Picture (intercept) | 33765.51 | 183.75 |  |  | 33761.05 | 183.74 |  |  |
| Fixed effects | *β* | *SE* | *t* | *p* | *β* | *SE* | *t* | *p* |
| Intercept | 1226.81 | 24.68 | 49.71 | < .001 | 1226.32 | 24.16 | 50.76 | < .001 |
| Cognate Status | -39.38 | 14.04 | -2.81 | .006 | -39.35 | 14.04 | -2.80 | .006 |
| List Type | -8.95 | 4.55 | -1.97 | .049 | -9.06 | 4.55 | -1.99 | .047 |
| L1 Stroop Congruency | -25.07 | 21.30 | -1.18 | .245 | -39.63 | 20.57 | -1.93 | .060 |
| L1 Stroop PC | -12.99 | 21.35 | -.61 | .546 | -13.16 | 20.71 | -.64 | .528 |
| Cognate Status × List Type | -3.15 | 4.56 | -.69 | .489 | -3.26 | 4.56 | -.71 | .475 |
| Cognate Status × L1 Stroop Congruency | 9.57 | 4.59 | 2.08 | .037 | 9.67 | 4.64 | 2.08 | .037 |
| Cognate Status × L1 Stroop PC | -5.81 | 4.63 | -1.25 | .210 | -7.15 | 4.63 | -1.54 | .123 |
| List Type × L1 Stroop Congruency | 4.15 | 4.60 | .90 | .367 | .03 | 4.65 | .01 | .995 |
| List Type × L1 Stroop PC | 3.21 | 4.63 | .69 | .488 | 1.91 | 4.63 | .41 | .681 |
| Cognate Status × List Type × L1 Stroop Congruency | -4.09 | 4.61 | -.89 | .374 | -6.17 | 4.65 | -1.33 | .184 |
| Cognate Status × List Type × L1 Stroop PC | 6.18 | 4.63 | 1.34 | .182 | 6.24 | 4.63 | 1.35 | .178 |

Figure S5

*The impact of the L1 Stroop congruency effect, expressed in raw RTs (A) and z-transformed RTs (B), on RTs for cognate and noncognate pictures in the L2 picture naming task*

*
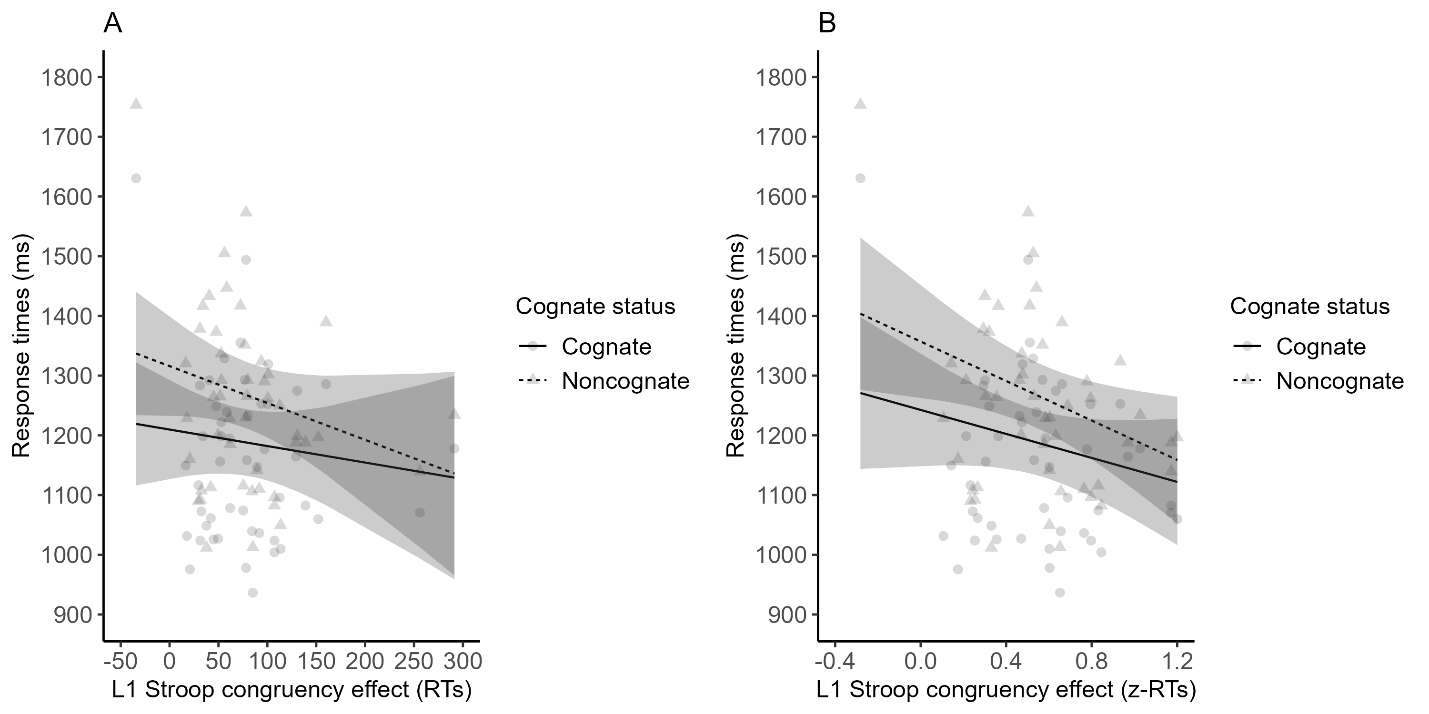
Note.* The circles and triangles represent mean participant RTs and the lines (with 95% confidence bands) represent the model-estimated trends for cognate and noncognate pictures.

Overall, this pattern appears to challenge current theories of language-control associations (e.g., Bialystok, 2017; Green & Abutalebi, 2013), theories which would seem to predict positive, not negative associations between performance on linguistic tasks and performance on conflict tasks. In contrast, what the present results seem to suggest is a trade-off between (linguistic) performance in the L2 and (processing-selectivity) performance in the L1: Individuals with greater ability to limit influences from their L1 (i.e., an Italian cognate or noncognate name) when using their L2 show reduced ability to respond using their L1 (i.e., naming in colors in Italian) in the presence of interfering information also presented in their L1 (i.e., incongruent Italian color words). In other words, individuals who perform better in their L2 seem to perform worse in their L1, what would appear to be a *dis*advantage (see, e.g., Runnqvist et al., 2013; Sandoval et al., 2010). Future research should attempt to probe the robustness of this pattern.

*List type order*

These analyses were conducted to determine whether the patterns involved in the present experiments might be modulated by the order in which participants received the two list types involved in the two tasks (i.e., mostly-cognate first vs. mostly-noncognate first for L2 picture naming and mostly-congruent first vs. mostly-incongruent first for L1 Stroop). Note that the List Type Order factor was counterbalanced across participants (even though it was always compatible across the two tasks, e.g., participants presented with the mostly-cognate list first in L2 picture naming were always presented with the mostly-congruent list first in L1 Stroop). Thus, our experiments were controlled for potential practice/fatigue effects. However, those effects can still arise and interact with those of the other variables being manipulated, for example, the basic congruency effect (e.g., MacLeod, 1998) and even the PC effect in the Stroop task (Abrahamse et al., 2013).

For L2 picture naming, the mean participant-based RTs and error rates are presented in Table S14. Full results from the RT and accuracy models are reported in Table S15. In the RTs, no new effect emerged in addition to the main effects of Cognate Status and List Type previously discussed. Notably, there was no effect of practice, which would have emerged as a significant interaction between List Type and List Type Order. No effect reached significance in the accuracy data, however, both the main effect of List Type Order and the interaction between List Type and List Type Order were marginal, reflecting numerical tendencies, respectively, for higher accuracy in participants who received the mostly-noncognate list first and for higher accuracy in the list that participants received as the second list (i.e., the mostly-noncognate list for participants who received the mostly-cognate list and the mostly-noncognate list for participants who received the mostly-noncognate list first), a practice effect.

Table S14

*Mean participant-based response times and percentage error rates (and corresponding 95% confidence intervals calculated using Cousineau’s (2019) method) in the L2 picture naming task, for participants who received the mostly-cognate list first and participants who received the mostly-noncognate list first*

|  | Response times | | Error rates | |
| --- | --- | --- | --- | --- |
| Cognate status | Mostly-cognate list | Mostly-noncognate list | Mostly-cognate list | Mostly-noncognate list |
| Mostly-cognate list first | | | | |
| Cognate | 1140 [1096, 1185] | 1169 [1128, 1210] | 11.83 [9.78, 13.88] | 11.54 [9.26, 13.82] |
| Noncognate | 1229 [1182, 1276] | 1254 [1209, 1298] | 14.53 [10.29, 18.78] | 12.63 [10.15, 15.11] |
| Cognate effect | 89 | 85 | 2.70 | 1.09 |
| Mostly-noncognate list first | | | | |
| Cognate | 1167 [1133, 1202] | 1170 [1129, 1211] | 9.71 [7.46, 11.95] | 10.44 [6.22, 14.67] |
| Noncognate | 1260 [1215, 1306] | 1251 [1211, 1291] | 9.52 [4.59, 14.44] | 12.13 [9.00, 15.26] |
| Cognate effect | 93 | 81 | -.19 | 1.69 |

Table S15

*Variances and standard deviations for the random effects and coefficients, standard errors, statistics, and probability values for the fixed effects used in the models of response times and accuracy in the L2 picture naming task adding the List Type Order predictor*

|  | Response times | | | | Accuracy | | | |
| --- | --- | --- | --- | --- | --- | --- | --- | --- |
| Random effects | *Variance* | *SD* |  |  | *Variance* | *SD* |  |  |
| Participant (intercept) | 20169.61 | 142.02 |  |  | .255 | .505 |  |  |
| Picture (intercept) | 33684.70 | 183.53 |  |  | 1.360 | 1.166 |  |  |
| Fixed effects | *β* | *SE* | *t* | *p* | *β* | *SE* | *z* | *p* |
| Intercept | 1227.05 | 24.84 | 49.40 | < .001 | 2.552 | .124 | 20.50 | < .001 |
| Cognate Status | -39.37 | 14.02 | -2.81 | .005 | .111 | .097 | 1.14 | .252 |
| List Type | -8.99 | 4.56 | -1.97 | .049 | .033 | .042 | .80 | .424 |
| List Type Order | -7.30 | 21.00 | -.35 | .730 | -.140 | .084 | -1.66 | .097 |
| Cognate Status × List Type | -3.19 | 4.56 | -.70 | .484 | -.008 | .042 | -.20 | .842 |
| Cognate Status × List Type Order | -.14 | 4.56 | -.03 | .976 | .030 | .042 | .73 | .466 |
| List Type × List Type Order | -6.56 | 4.56 | -1.44 | .151 | -.078 | .042 | -1.87 | .062 |
| Cognate Status × List Type × List Type Order | .38 | 4.56 | .08 | .934 | .053 | .042 | 1.28 | .201 |

*Note.* The accuracy coefficients are in the logit scale, not in the response scale.

For L1 Stroop, the mean participant-based RTs and error rates are presented in Table S16. Note that the 95% confidence intervals could not be calculated for participants who received the mostly-incongruent list first because there was no variability in one of the cells of the design for those participants (i.e., no errors were committed for congruent stimuli in the mostly-congruent list). Full results from the RT and accuracy models are reported in Table S17. Once again, the accuracy model failed to converge initially, although in this case it did not converge after being restarted from the apparent optimum either. What is reported is the model that we fit using the BOBYQA optimizer instead of lme4’s default optimizer, a model which did manage to converge. Further, note that, as with the other analyses with additional predictors, we did not include the random intercept for colors in that model because the previous analyses suggested that there was little variance associated with that intercept in our accuracy data.

No effect was significant in the accuracy data. In contrast, in the RTs, all effects except for the main effect of List Type Order were significant. First, there were the main effect of Congruency, the main effect of List Type, and their interaction described above. Further, there was an interaction between List Type and List Type Order reflecting overall faster responses in the list that participants received as the second list (i.e., the mostly-noncognate list for participants who received the mostly-cognate list and the mostly-noncognate list for participants who received the mostly-noncognate list first), a practice effect. There was also an interaction between Congruency and List Type Order, reflecting the fact that participants who received the mostly-congruent list first produced overall smaller congruency effects than participants who received the mostly-incongruent list first. The three-way interaction was also significant, with follow-up analyses revealing that the source of the interaction was that participants who received the mostly-congruent list first produced a smaller congruency effect than participants who received the mostly-incongruent list first only in the mostly-congruent list, *β* = 40.3, *SE* = 8.49, *z* = 4.74, *p* < .001, whereas the same tendency remained marginal in the mostly-incongruent list, and *β* = 15.8, *SE* = 8.47, *z* = 1.87, *p* = .061. As a result, the PC effect was smaller in participants who received the mostly-congruent vs. mostly-incongruent list first, although both groups showed the effect (*β* = -21.6, *SE* = 8.47, *z* = -2.55, *p* = .011 and *β* = -46.1, *SE* = 8.49, *z* = -5.43, *p* < .001, respectively). Note that this pattern is the opposite of the one documented by Abrahamse et al. (2013), i.e., a smaller PC effect in participants who received the mostly-*incongruent* list first. However, that pattern was not without problems given that the experiment that produced it, unlike the present one, involved no control for non-conflict processes, among other things (Schmidt, 2016). Further, we have not always been able to reproduce it in other works of ours (Spinelli et al., in preparation; Spinelli & Lupker, 2023). The present pattern contributes to this mixed set of findings. The most reasonable interpretation, at this point, might be that participants who received the mostly-congruent list first in our experiment happened to be a bit better than those who received the mostly-incongruent list first at selecting the color ignoring the word. The implication is that they would show reduced congruency effects, especially in the list that normally tends to inflate those effects, i.e., the mostly-congruent list. As a result, the PC effect would be reduced in those participants (for similar patterns see, e.g., Hutchison, 2011; Kane & Engle, 2003). In any case, the fact that both participants who received the mostly-congruent list first and those who received the mostly-incongruent list first showed a PC effect is a testament to the robustness of this effect.

Table S16

*Mean participant-based response times and percentage error rates (and corresponding 95% confidence intervals calculated using Cousineau’s (2019) method) in the L1 Stroop task, for participants who received the mostly-congruent list first and participants who received the mostly-incongruent list first*

|  | Response times | | Error rates | |
| --- | --- | --- | --- | --- |
| Congruency | Mostly-congruent list | Mostly-incongruent list | Mostly-congruent list | Mostly-incongruent list |
| Mostly-congruent list first | | | | |
| Congruent | 739 [715, 762] | 720 [700, 740] | .61 [.01, 1.21] | .43 [-.22, 1.09] |
| Incongruent | 815 [791, 839] | 774 [750, 798] | 2.02 [.90, 3.13] | 1.83 [.73, 2.94] |
| Congruency effect | 76 | 54 | 1.41 | 1.40 |
| Mostly-incongruent list first | | | | |
| Congruent | 660 [633, 686] | 690 [667, 713] | 0 | .17 |
| Incongruent | 777 [746, 808] | 761 [729, 792] | 4.44 | 2.29 |
| Congruency effect | 117 | 71 | 4.44 | 2.12 |

Table S17

*Variances and standard deviations for the random effects and coefficients, standard errors, statistics, and probability values for the fixed effects used in the models of response times and accuracy in the L1 Stroop task adding the List Type Order predictor*

|  | Response times | | | | Accuracy | | | |
| --- | --- | --- | --- | --- | --- | --- | --- | --- |
| Random effects | *Variance* | *SD* |  |  | *Variance* | *SD* |  |  |
| Participant (intercept) | 11239.70 | 106.02 |  |  | .275 | .525 |  |  |
| Color (intercept) | 6.61 | 2.57 |  |  |  |  |  |  |
| Fixed effects | *β* | *SE* | *t* | *p* | *β* | *SE* | *z* | *p* |
| Intercept | 741.50 | 15.43 | 48.06 | < .001 | 6.629 | 4.071 | 1.63 | .103 |
| Congruency | -39.50 | 1.50 | -26.35 | < .001 | 2.825 | 4.070 | .69 | .488 |
| List Type | 5.59 | 1.50 | 3.73 | < .001 | 1.614 | 4.070 | .40 | .692 |
| List Type Order | 20.33 | 15.38 | 1.32 | .193 | -1.927 | 4.071 | -.47 | .636 |
| Congruency × List Type | -8.46 | 1.50 | -5.64 | < .001 | 1.814 | 4.070 | .45 | .656 |
| Congruency × List Type Order | 7.01 | 1.50 | 4.68 | < .001 | -2.160 | 4.070 | -.53 | .596 |
| List Type × List Type Order | 9.12 | 1.50 | 6.09 | < .001 | -1.724 | 4.070 | -.42 | .672 |
| Congruency × List Type × List Type Order | 3.05 | 1.50 | 2.04 | .042 | -1.874 | 4.070 | -.46 | .645 |

*Note.* The accuracy coefficients are in the logit scale, not in the response scale.

**References (not already included in the main text’s reference list)**

Abrahamse, E. L., Duthoo, W., Notebaert, W., & Risko, E. F. (2013). Attention modulation by proportion congruency: The asymmetrical list shifting effect. *Journal of Experimental Psychology: Learning, Memory, and Cognition*, *39*, 1552–1562. https://doi.org/10.1037/a0032426

Bar-Ilan, L., & Berman, R. A. (2007). Developing register differentiation: the Latinate-Germanic divide in English. *Linguistics*, *45*(1), 1–35. https://doi.org/10.1515/ling.2007.001

Barr, D. J., Levy, R., Scheepers, C., & Tily, H. J. (2013). Random effects structure for confirmatory hypothesis testing: Keep it maximal. *Journal of Memory and Language*, *68*(3), 255–278. https://doi.org/10.1016/j.jml.2012.11.001

Bates, D., Kliegl, R., Vasishth, S., & Baayen, H. (2015a). Parsimonious mixed models. *ArXiv*, 1506.04967. https://doi.org/10.48550/arXiv.1506.04967

Bates, D., Mächler, M., Bolker, B., & Walker, S. (2015b). Fitting linear mixed-effects models using lme4. *Journal of Statistical Software*, *67*(1), 1–48. https://doi.org/10.18637/jss.v067.i01

de Leeuw, J. R. (2015). jsPsych: A JavaScript library for creating behavioral experiments in a Web browser. *Behavior Research Methods*, *47*(1), 1–12. https://doi.org/10.3758/s13428-014-0458-y

Hilchey, M. D., & Klein, R. M. (2011). Are there bilingual advantages on nonlinguistic interference tasks? Implications for the plasticity of executive control processes. *Psychonomic Bulletin & Review*, *18*(4), 625–658. https://doi.org/10.3758/s13423-011-0116-7

Hilchey, M. D., Saint-Aubin, J. & Klein, R. M. (2015) Does bilingual exercise enhance cognitive fitness in traditional non-linguistic executive processing tasks? In J. W. Schwieter (Ed.), *The Cambridge handbook of bilingual processing* (pp. 586-613). Cambridge, UK: Cambridge University Press. https://doi.org/10.1017/cbo9781107447257.026

Hutchison, K. A. (2011). The interactive effects of listwide control, item-based control, and working memory capacity on Stroop performance. *Journal of Experimental Psychology: Learning, Memory, and Cognition*, *37*, 851–860. https://doi.org/10.1037/a0023437

Kane, M. J., & Engle, R. W. (2003). Working-memory capacity and the control of attention: the contributions of goal neglect, response competition, and task set to Stroop interference. *Journal of Experimental Psychology: General*, *132*, 47–70. https://doi.org/10.1037/0096-3445.132.1.47

Kuznetsova, A., Brockhoff, P. B., & Christensen, R. H. B. (2017). lmerTest Package: Tests in Linear Mixed Effects Models. *Journal of Statistical Software*, *82*(13), 1–26. https://doi.org/10.18637/jss.v082.i13

Lenth, R. (2022). Emmeans: Estimated Marginal Means, aka Least-Squares Means. R package version 1.7.5. https://CRAN.R-project.org/package=emmeans

MacLeod, C. M. (1998). Training on integrated versus separated Stroop tasks: The progression of interference and facilitation. *Memory & Cognition*, *26*, 201–211. https://doi.org/10.3758/BF03201133

Morey, R., & Rouder, J. (2022). BayesFactor: Computation of Bayes Factors for common designs. R package version 0.9.12-4.4. https://CRAN.R-project.org/package=BayesFactor

Paap, K. R. (2018). Bilingualism in cognitive science: The characteristics and consequences of bilingual language control. In A. De Houwer & L. Ortega (Eds.), *The Cambridge handbook of bilingualism* (pp. 435– 465). Cambridge, UK: Cambridge University Press. https://doi.org/10.1017/9781316831922.023

Paap, K. R., & Greenberg, Z. I. (2013). There is no coherent evidence for a bilingual advantage in executive processing. *Cognitive Psychology*, *66*(2), 232–258. https://doi.org/10.1016/j.cogpsych.2012.12.002

Runnqvist, E., Gollan, T. H., Costa, A., & Ferreira, V. S. (2013). A disadvantage in bilingual sentence production modulated by syntactic frequency and similarity across languages. *Cognition*, *129*(2), 256–263. https://doi.org/10.1016/j.cognition.2013.07.008

Sandoval, T. C., Gollan, T. H., Ferreira, V. S., & Salmon, D. P. (2010). What causes the bilingual disadvantage in verbal fluency? The dual-task analogy. Bilingualism: Language and Cognition, 13(2), 231–252. https://doi.org/10.1017/s1366728909990514

Schmidt, J. R. (2016). Proportion congruency and practice: A contingency learning account of asymmetric list shifting effects. *Journal of Experimental Psychology: Learning, Memory, and Cognition*, *42*, 1496–1505. https://doi.org/10.1037/xlm0000254

Spinelli, G., Ambrosini, E., Vallesi, A., & Lupker, S. J. (in preparation). Adaptive control in Stroop, Stroop-like, and Simon tasks.

Spinelli, G., Goldsmith, S. F., Lupker, S. J., & Morton, J. B. (2022). Bilingualism and executive attention: Evidence from studies of proactive and reactive control. *Journal of Experimental Psychology: Learning, Memory, and Cognition*, *48*(6), 906–927. https://doi.org/10.1037/xlm0001095

Sulpizio, S., Del Maschio, N., Del Mauro, G., Fedeli, D., & Abutalebi, J. (2020). Bilingualism as a gradient measure modulates functional connectivity of language and control networks. *NeuroImage*, *205*, 116306. https://doi.org/10.1016/j.neuroimage.2019.116306

Tomoschuk, B., Ferreira, V. S., & Gollan, T. H. (2019). When a seven is not a seven: Self-ratings of bilingual language proficiency differ between and within language populations. *Bilingualism: Language and Cognition*, *22*(3), 516–536. https://doi.org/10.1017/s1366728918000421
